# A New 13 Million Year Old Gavialoid Crocodylian from Proto-Amazonian Mega-Wetlands Reveals Parallel Evolutionary Trends in Skull Shape Linked to Longirostry

**DOI:** 10.1371/journal.pone.0152453

**Published:** 2016-04-20

**Authors:** Rodolfo Salas-Gismondi, John J. Flynn, Patrice Baby, Julia V. Tejada-Lara, Julien Claude, Pierre-Olivier Antoine

**Affiliations:** 1 Institut des Sciences de l’Évolution, Université de Montpellier, CNRS, IRD, EPHE, 34095, Montpellier, France; 2 Departamento de Paleontología de Vertebrados, Museo de Historia Natural, Universidad Nacional Mayor de San Marcos; Avenida Arenales 1256, Lima 14, Perú; 3 Division of Paleontology, American Museum of Natural History, New York, NY, 10024–5192, United States of America; 4 Géosciences-Environnements Toulouse, Université de Toulouse; UPS (SVT-OMP); CNRS; IRD; 14 Avenue Édouard Belin, F-31400, Toulouse, France; 5 Convenio IRD-PeruPetro, Av. Luis Aldana 320, San Borja, Lima, Perú; 6 Department of Earth and Environmental Sciences, Columbia University, New York City, New York, United States of America; Team 'Evolution of Vertebrate Dentition', FRANCE

## Abstract

Gavialoid crocodylians are the archetypal longirostrine archosaurs and, as such, understanding their patterns of evolution is fundamental to recognizing cranial rearrangements and reconstructing adaptive pathways associated with elongation of the rostrum (longirostry). The living Indian gharial *Gavialis gangeticus* is the sole survivor of the group, thus providing unique evidence on the distinctive biology of its fossil kin. Yet phylogenetic relationships and evolutionary ecology spanning ~70 million-years of longirostrine crocodylian diversification remain unclear. Analysis of cranial anatomy of a new proto-Amazonian gavialoid, *Gryposuchus pachakamue* sp. nov., from the Miocene lakes and swamps of the Pebas Mega-Wetland System reveals that acquisition of both widely separated and protruding eyes (telescoped orbits) and riverine ecology within South American and Indian gavialoids is the result of parallel evolution. Phylogenetic and morphometric analyses show that, in association with longirostry, circumorbital bone configuration can evolve rapidly for coping with trends in environmental conditions and may reflect shifts in feeding strategy. Our results support a long-term radiation of the South American forms, with taxa occupying either extreme of the gavialoid morphospace showing preferences for coastal marine versus fluvial environments. The early biogeographic history of South American gavialoids was strongly linked to the northward drainage system connecting proto-Amazonian wetlands to the Caribbean region.

## Introduction

Numerous unresolved issues hinder understanding of the origin, time of divergence, and patterns of adaptive radiation of gavialoid crocodylians. Whereas molecular data sets favor a close relationship and an Eocene [1] or even Neogene [2–4] divergence between the Indian gharial *Gavialis gangeticus* and the Indonesian false gharial *Tomistoma schlegelii*, morphological phylogenies suggest that: 1) these two extant longirostrine species are much more distantly related, 2) their elongated skulls evolved convergently, and 3) the oldest fossil gavialoid dates back to the Cretaceous Period in North America, Europe, and Africa [5,6]. In fact, analyses of extant *Gavialis* and its nearest fossil relatives do not provide strong support for their phylogenetic affinities with any other crocodylian clade, probably as part of what Clark [7] called the “longirostrine problem”. Clark [7] identified several cranial features related to longirostry in crocodyliforms that might have appeared independently in different groups, including possessing widely separated and protruding eyes, namely “telescoped” orbits. Potential convergent evolution of this distinctive cranial morphotype in gavialoids not only obscured the ancestral anatomical condition for the entire clade, but also created a challenge for deciphering ingroup affinities. For example, Indian *Gavialis* and South American *Gryposuchus* species are extremely similar in cranial morphology and both have fully “telescoped” orbits [8,9]. Whether this distinctive pattern results from common or independent origin has been uncertain and debated [6,10]. Here, we describe a gavialoid with “non-telescoped” orbits from the Middle Miocene of the Pebas Formation of northeastern Peru, that provides evidence of early morphological stages of the evolution of the *Gryposuchus* lineage in the Amazonian Neotropics relevant to resolving shared or independent origin of “telescoped” orbits. This new gavialoid is the only longirostrine species in the hyperdiverse crocodylian community that inhabited lakes, swamps, and deltas of the Pebas Mega-Wetland System [11], a huge proto-Amazonian biome that appears to have played a crucial role for marine to freshwater transitions in many vertebrate groups occurring today in various river drainage systems of tropical South America [12]. Its lineage survived within the paleo-Orinoco drainage throughout the Late Miocene, providing further evidence for the persistence of Pebas-like mega-wetland conditions in aquatic environments of northernmost South America [11]. These records highlight the biogeographic role of the long-lasting drainage linking western proto-Amazonia with the Caribbean region prior to onset of the eastward flowing, transcontinental Amazonian River system approximately 10.5 million years ago. We analyze the phylogenetic relationships of this new Pebasian species to test whether parallel evolution occurred within an adaptive radiation of Indian and South American gavialoids. Mapping our phylogenetic hypothesis onto a morphometric space, the pattern of the remarkable taxonomic and anatomical diversification of South American forms provides novel insights into the ecological significance underlying circumorbital skull configurations throughout gavialoid history.

## Materials and Methods

The Natural History Museum of National University of San Marcos (MUSM), Lima, Peru, provided all permits for this study and houses permanently the specimens from the Pebas Formation described in this publication (Credential Number MUSM 2008–8). All data has been collected, prepared and deposited in this public national Peruvian repository by the authors of the article. The current data is based only in fossil specimens, to which it has been assigned a specific catalog number. Specimen numbers are MUSM 1981, MUSM 987, MUSM 900, MUSM 1681, MUSM 2032, MUSM 1988, MUSM 1727, MUSM 1439, MUSM 2407, MUSM 1428, MUSM 1435, MUSM 1440, MUSM 1682, MUSM 1993, MUSM 2470, MUSM 2471, MUSM 2472. All of these Peruvian fossil specimens are completely available in this public repository. The context information for each fossil specimen is saved in an internal database of the repository institution.

### Phylogenetic analysis

To determine the phylogenetic relationships of the new Pebasian gavialoid species, we included it in a data matrix of morphological characters revised and updated from Salas-Gismondi et al. [11], that built on characters initially developed by Brochu [13,14] and Jouve [6], as well as additional characters compiled from other contributions (S1 Appendix). The present analysis focuses on testing the phylogenetic relationships among gavialoid crocodylians rather than more broadly across all Crocodylia as in the former analyses. The complete data matrix consists of 206 morphological characters for 42 eusuchian taxa, with *Bernissartia fagesii* as an outgroup and including most members of the gavialoid clade but only representative taxa of Brevirostres (S1 Appendix). This matrix was analyzed with maximum parsimony methods using TNT 1.1 [15]. All characters had equal weighting and were treated as unordered and non-additive. To assess nodal support, branches with a minimum length of 0 were collapsed and Bremer support values (decay indices) were calculated and shown on the strict consensus phylogeny.

### Cranial circumorbital morphospace analysis and phylogenetic mapping

In order to understand morphological evolution linked to longirostry, we applied a geometric morphometric approach to analysis of the circumorbital anatomy of 22 species of crocodylians, including extant and extinct species (S1 Appendix). As the circumorbital region has shown only minor intraspecific variation within extant adult crocodylians [16], all taxa are represented by one fully adult specimen, each preserving in dorsal view: (1) rostral sutures, (2) orbital shape pattern, and (3) no significant postmortem distortion. Morphometric analyses require complete datasets for all landmarks, thus MUSM 1981 was partially reconstructed from the morphology of the complete left side via geometric reflection [17], and included in the analysis. We selected twenty discrete landmark loci based on their availability, relative co-planarity, and clear demarcation within images (S1 Fig). Two-dimensional landmark digitization, Procrustes superimposition, and principal component analyses (PCA) were performed with the Geomorph package in R software [18, 19]. The 22 landmark configurations were superimposed and scaled to unit centroid size following the generalized Procrustes method [20]. The coordinates of the superimposed configurations were later imported into the Euclidean tangent shape space for subsequent statistical analysis [20]. In order to determine components of shape variation, the 22 principal components from that analysis were obtained by applying a principal component analysis (PCA) on the variance covariance of the projected coordinates. Principal component axes (PCs) 1 and 2 (estimated cumulative variance = 70.6%) were plotted in principal components space. A simplified version of the parsimony-based phylogenetic hypothesis unweighted for branch length and including only the taxa sampled for the twenty landmarks, was mapped in the circumorbital morphospace with MorphoJ software [21].

### Nomenclatural acts

The electronic edition of this article conforms to the requirements of the amended International Code of Zoological Nomenclature, and hence the new names contained herein are available under that Code from the electronic edition of this article. This published work and the nomenclatural acts it contains have been registered in ZooBank, the online registration system for the ICZN. The ZooBank LSIDs (Life Science Identifiers) can be resolved and the associated information viewed through any standard web browser by appending the LSID to the prefix “http://zoobank.org/”. The LSID for this publication is: urn:lsid:zoobank.org:pub:31C1EBEF-A7B1-44BA-ACB4-1552AD2BC650. The electronic edition of this work was published in a journal with an ISSN, and has been archived and is available from the following digital repositories: PubMed Central, LOCKSS.

## Geological Context, Paleoenvironment, and Age

**Fig 1:**
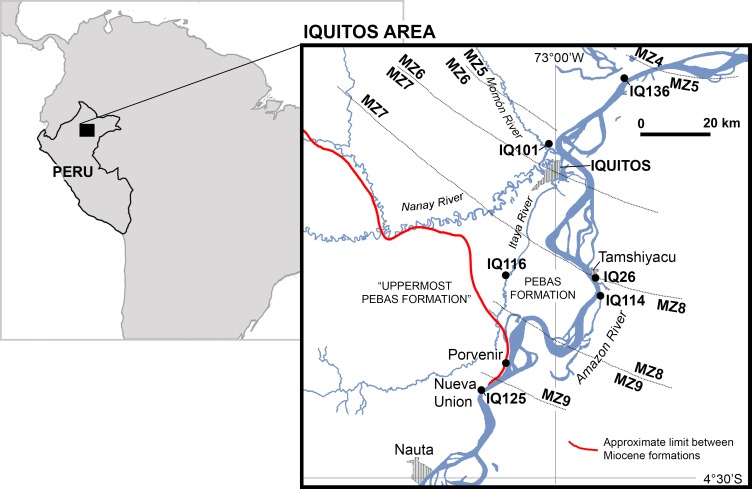
Miocene fossiliferous localities in the Iquitos region, northeastern Peru. Spatial distribution of the Molluscan Zones (MZ) [28] and fossil bearing localities (IQ) within the Pebas Formation and the “Uppermost Pebas Formation” is shown. Geological data from [33].

In the study area, the Pebas Formation is comprised of transgressive and regressive bay-margin deposits. Parasequences are formed by fossiliferous blue to gray clays interbedded with unconsolidated sands, which are typically capped by lignite layers and mollusc shell beds [22–24]. Lacustrine brackish-water, sometimes tidally influenced, mega-wetland deposits are indicated by most of the studies in the lower and middle parts of the Pebas Formation. The time span of the Pebas Formation ranges approximately from the early Miocene (ca. 23 Ma) to the early Late Miocene (ca. 10.5 Ma) [25]. The biostratigraphic framework of the Pebas Formation is based on pollen, ostracod, and particularly on the abundant and diverse molluscan faunas preserved throughout the unit [26–28]. Fossiliferous deposits are located along the Amazon River banks and tributaries of the so-called Iquitos Arch, which corresponds to the modern forebulge of the northwestern Amazonia foreland basin [24]. Fossil vertebrates are found mainly in lignitic bonebeds [11]. These localities (IQ) are mapped within the Molluscan Zones (MZ) proposed by Wesselingh et al., [28] for the study area (Fig 1). Most of the gavialoid remains come from localities IQ26 and IQ114, and correspond to MZ8 (late Middle Miocene; ca. 13 Ma). These localities have documented the highest diversity within any single known extant or fossil crocodylian community, including the shoveling mollusc-crushing caiman *Gnatusuchus pebasensis*, as well as *Kuttanacaiman iquitosensis*, *Caiman wannlangstoni*, *Purussaurus neivensis*, *Mourasuchus atopus*, *Paleosuchus* sp. [11], and the new gavialoid taxon described here. Other fossil vertebrate remains, such as fishes, aquatic turtles, and mammals, also are abundant. IQ116 is a less productive locality than IQ26 and IQ114, but might correspond to the same time interval. This later outcrop is restricted to a 1–2 meter thick capping lignite level overlying a grey mud with shell beds [11]. Two specimens of the new Pebasian gavialoid, MUSM 1681 and MUSM 1682, were found in locality IQ136, probably corresponding to MZ5 (Middle Miocene; ca. 16–15 Ma). These are the oldest gavialoids known from the Amazon Basin. An additional gavialoid specimen, MUSM 987 was unearthed at IQ101, in the Momón River banks, and might correspond in age to MZ6 (ca. 15–14 Ma). Pebas Formation deposits represent a highly dynamic mosaic of shallow aquatic settings (e.g., lakes, swamps, and rivers) of varying salinity and marine influence [29–32], and dysoxic muddy bottoms within lakes and swamps were common in the Pebas System. Several gavialoid bones, including a diagnostic postorbital bone (MUSM 2430) belonging to *Gryposuchus colombianus* or *Gr*. *croizati*, were found at locality IQ125 in the Nueva Unión area. This locality belongs to the “Uppermost Pebas Formation” (Fig 1) [11,33] and might correspond to MZ9 or younger intervals (Middle-Late Miocene boundary; ca. 12–11 Ma). Outcrops there consist of fine-grained fluvial sandstones, floodplain clays and silts, and paleosols. These levels are lignite-poor and lack mollusc remains [33].

**Fig 2:**
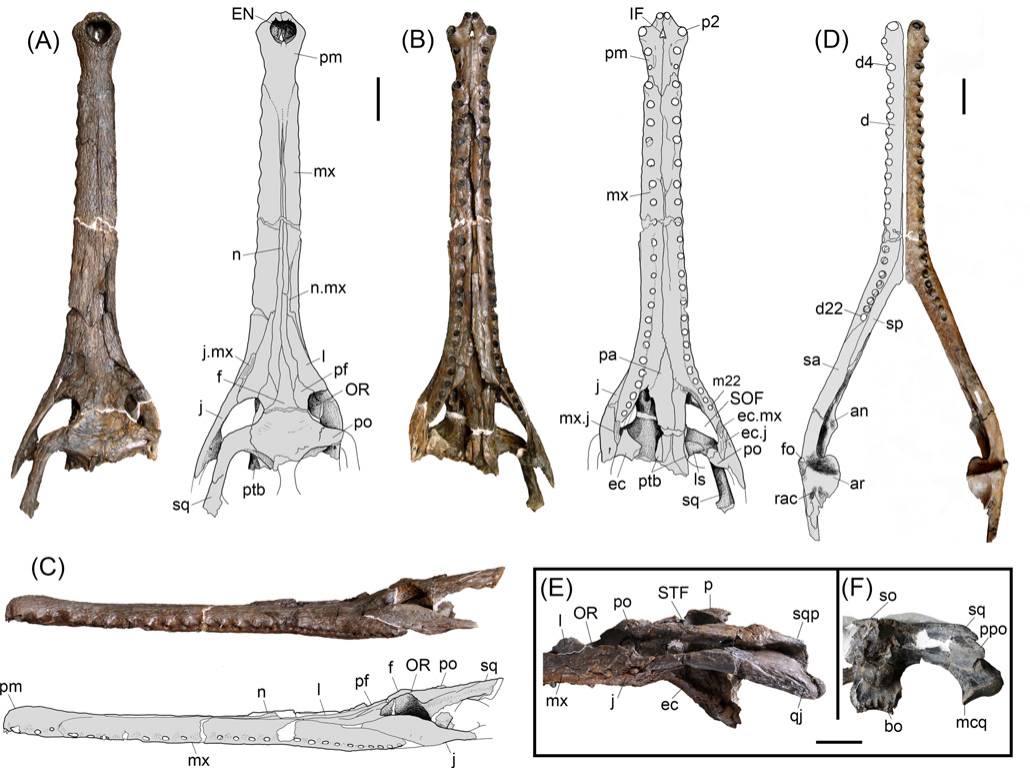
*Gryposuchus pachakamue* sp. nov. Photograph and schematic drawing of the skull (holotype, MUSM 1981) in dorsal (A), ventral (B), and lateral (C) view. (D) Photograph of the right mandible (MUSM 987) and schematic drawing in dorsal view. Details of the skull (E,F). (E) MUSM 900 in lateral view. (F) MUSM 1681 in occipital view. Abbreviations: an, angular; ar, articular; bo, basioccipital; d, dentary; d4, d22, dentary tooth positions; ec, ectopterygoid; ec.j, jugal surface for ectopterygoid; ec.mx, maxilla surface for ectopterygoid; EN, external nares; eo, exoccipital; f, frontal; fo, foramen; IF, incisive foramen; j, jugal; j.mx, maxilla surface for the jugal; l, lacrimal; ls, laterosphenoid; m22, maxillary tooth position 22; mcq, medial condyle of the quadrate; mx, maxilla; mx.j, jugal surface for maxilla; n.mx, maxilla surface for nasal; n, nasal; OR, orbit; p, parietal; pa, palatine; pf, prefrontal; pm, premaxilla; p2, premaxillary tooth position 2; po, postorbital; ppo, paraoccipital process; pt, pterygoid; ptb, pterygoid bullae; q, quadrate; qj, quadratojugal; qj.q, quadrate surface for quadratojugal;; rac, retroarticular crest; sp, splenial; sa, surangular; so, supraoccipital; sq, squamosal; SOF, suborbital fenestra; STF, supratemporal fenestra. Scale bars, 5 cm.

Among the copious gavialoid material reported from the Late Miocene Urumaco Formation of Venezuela, we identified a taxon consistent in morphology with the new Pebasian gavialoid [34]. The Urumaco Formation consists of complex intercalation of sandstone, organic-rich mudstone, coal, shale, and thick-bedded coquinoidal limestones with abundant mollusc fragments [35]. The Urumaco specimen comprises a partial skull (AMU CURS 12; Fig 2D) collected within the upper member of the Urumaco Formation, at the Domo de Agua Blanca Locality [34]. This member is characterized by organic-rich, dark-gray laminated mudstone and shale, and abundant vertebrate fragments [35]. The upper member of the Urumaco Formation was deposited in a delta plain [35].

## Results

### Systematic paleontology

Crocodyliformes Hay, 1930 [36]

Eusuchia Huxley, 1875 [37]

Crocodylia Gmelin, 1789 [38]

Gavialoidea Hay, 1930 [36]

Gryposuchinae Vélez-Juarbe et al., 2007 [39]

*Gryposuchus* Gürich, 1912 [40]

*Gryposuchus pachakamue* sp. nov.

ZooBank life science identifier (LSID) for species. urn:lsid:zoobank.org:act:6B71903E-9412-44BE-8537-203B07909DEE

#### Etymology

The Pebasian *Gryposuchus* species is named after the Quechua word “*pachakamue*”, primordial pre-Columbian god and first “storyteller” [41] who preserved ancient knowledge about the origin of living things in Amazonia.

#### Holotype

MUSM 1981, nearly complete skull (Fig 1A–1C).

#### Locality and Horizon

Locality IQ114, Iquitos area, Peru; Pebas Formation, late Middle Miocene, approx. 13 Ma; Mollusc Zone 8 (MZ8; Fig 1) [28].

#### Referred specimens

**Fig 3:**
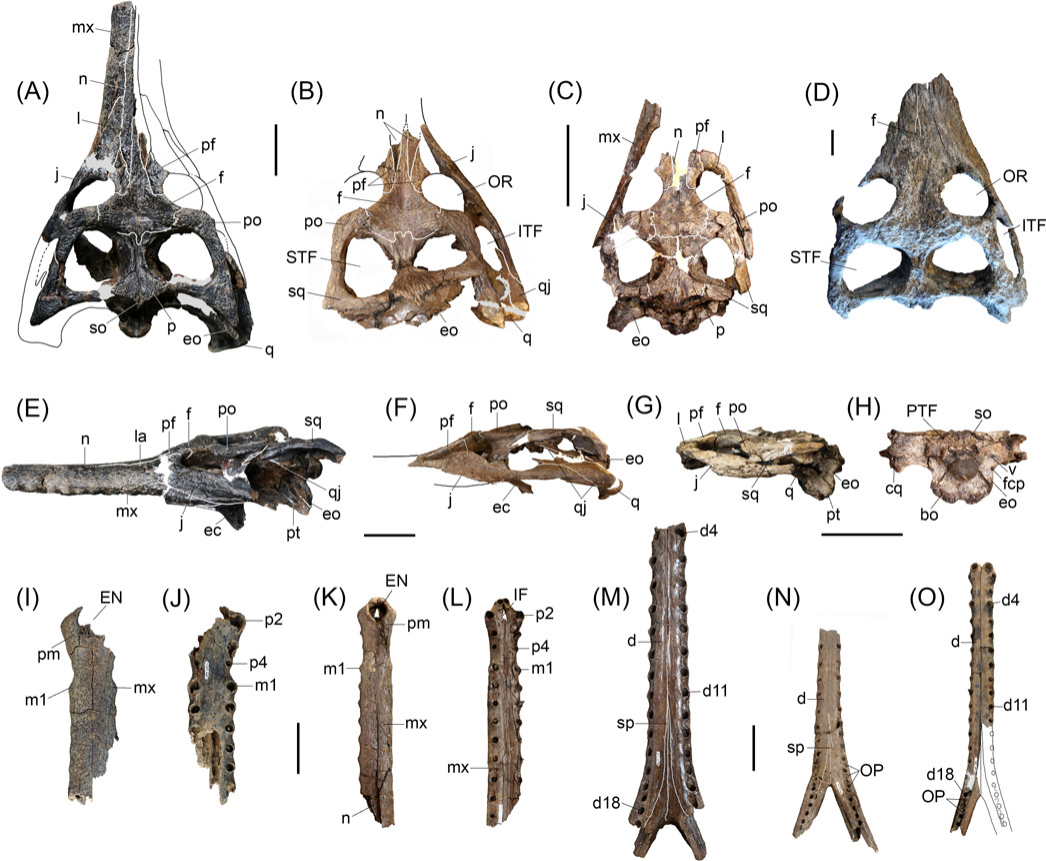
Cranial and mandibular specimens referred to *Gryposuchus pachakamue* sp. nov. (A*-*C,E-O) *Gryposuchus pachakamue* from the Middle Miocene of the Pebas Formation, Peru and (D) *Gryposuchus* cf. *pachakamue* from the Late Miocene of Urumaco, Venezuela. (A,E) Skull in dorsal (A) and lateral (E) view (MUSM 1681); (B,F) Skull in dorsal (B) and lateral (F) view (MUSM 2032); (C,G,H) Skull in dorsal (C), lateral (G), and occipital (H) view, juvenile (MUSM 1988); (D) Skull in dorsal view (AMU CURS 12). (I,J) Snout in dorsal (I) and (J) ventral view (MUSM 1681). (K,L) Snout in dorsal (K) and ventral (L) view, juvenile (MUSM 1727). (M) Symphyseal mandible in dorsal view (MUSM 2407). (N) Symphyseal mandible in dorsal view, juvenile (MUSM 1439). (O) Symphyseal mandible in dorsal view, juvenile (MUSM 1682). Abbreviations: cqg, cranio-quadrate groove; d, dentary; d4, d11, d18, dentary tooth positions; ec, ectopterygoid; EN, external nares; eo, exoccipital; f, frontal; fcp, foramen carotideum posterior; IF, incisive foramen; ITF, infratemporal fenestra; j, jugal; l, lacrimal; m1, maxillary tooth position 1; mx, maxilla; n, nasal; OP, occlusal pits; OR, orbit; p, parietal; p1, p2, p4, premaxillary tooth positions; pm, premaxilla; po, postorbital; pr, prefrontal; pt, pterygoid; PTF, post-temporal fenestra; q, quadrate; qj, quadratojugal; so, supraoccipital; sp, splenial; sq, squamosal; STF, supratemporal fenestra; v, foramen vagus. Scale bars, 5 cm.

MUSM 987, right mandible, Locality IQ101 (MZ6; Fig 2D); MUSM 900, skull without anterior half of the snout, Locality IQ116 (MZ8; Fig 2E); MUSM 1681, partial skull, Locality IQ136 (MZ5; Fig 2F and Fig 3A, 3I and 3J); MUSM 1682, symphyseal mandible of a juvenile, Locality IQ136 (MZ5; Fig 3O); MUSM 2032, skull without the snout, Locality IQ116 (MZ8; Fig 3B); MUSM 1988; skull table with orbital region of a juvenile, Locality IQ114 (MZ8; Fig 3C, 3G and 3H); MUSM 1435, symphyseal mandible, Locality IQ114; Fig 3M); MUSM 1727, snout of a juvenile, Locality IQ26 (MZ8; Fig 3K and 3L); MUSM 1439 symphyseal mandible of a juvenile, Locality IQ26 (MZ8; Fig 3N).

#### Diagnosis

*Gryposuchus pachakamue* is a long-snouted gavialoid crocodylian diagnosed by the following unique combination of characters: 22 maxillary and 22 mandibular teeth; ventral margin of postorbital bar inset from lateral jugal surface; frontoparietal suture between supratemporal fenestrae strongly concavoconvex; splenial with anterior perforation for mandibular ramus of cranial nerve V posterior to symphysis; splenial constricted within symphysis; surangular-dentary suture intersecting external mandibular fenestra at posterodorsal corner; surangular-articular suture bowed strongly laterally within glenoid fossa. Differs from *Gr*. *croizati* and *Gr*. *colombianus* in having nasals and premaxillae in extensive contact (character 82–2), narial opening longer than wide (character 83–0), posterior orbital margin not upturned (character 137–1), ventral margin of the orbit gently circular (character 138–0), and interorbital bridge width equivalent to orbit width (character 190–0).

### Description

The type of *Gryposuchus pachakamue* (MUSM 1981; Table 1) is a well-preserved skull, only slightly distorted in the right orbital region. It lacks most of the temporal and occipital bones, as well as the pterygoid flanges. The skull table is incomplete in the holotype, but well preserved in the referred specimens MUSM 1681 (Table 1) and MUSM 2032, and partially distorted in MUSM 900 (Table 1). The preserved length (from tip of the snout to the posterior angle of left supratemporal fenestra) of the type skull is 623.2 mm (Fig 1A–1C). Other referred specimens (MUSM 1440, MUSM 987, MUSM 2032, MUSM 1428, MUSM 1681, MUSM 2407, MUSM 2472, MUSM 2470, and MUSM 2471; Table 2), including cranial and mandibular remains, represent individuals of equivalent size to the holotype with the exception of MUSM 900, which is a larger skull lacking the anterior half of the snout (Fig 1E). In contrast, MUSM 1439, MUSM 1727, MUSM 1993, MUSM 1682, and MUSM 1988 comprise specimens that are notably smaller in size, with features typically associated with juvenile morphology, and herein referred to *Gr*. *pachakamue* (Fig 3C, 3G and 3H) as likely juvenile individuals. The description of the mandible is mainly based on MUSM 987, a complete right mandible corresponding to an individual of equivalent size to the holotype (Fig 1D). This specimen only lacks the coronoid bone and posterior end of the retroarticular process. Parts of the posterior lamina of the splenial that are in contact with the surangular are missing or collapsed. No postcranial material has been not definitively identified as pertaining to this new species.

**Table 1:** Measurements of the holotype and representative referred cranial specimens of *Gryposuchus pachakamue*.

	MUSM 1981	MUSM 1681	MUSM 900
Basal length of the skull	—	—	—
Greatest width	—	—	291.8
Width of the rostrum, posterior	125.2e	—	160.5e
Length of the snout, medial axe	463.1	—	—
Length of skull, dorsal	—	—	—
Interorbital distance	51.2	42.1	59.0
Orbit length	44.3e	34.0e	58.3e
Skull table width, anterior	149.0e	144.8	171.3
Skull table length, lateral	—	129.5	172.4
Skull table width, posterior	—	207.9	262.1
Skull width across postorbital bars	—	—	231.6
Occipital condyle width	—	34.5	36.3
Occipital condyle height	—	24.3	28.3
Orbit width	56.8(l)	54.0e (l)	63.6(r)
Nares width	28.3	—	—
Nares length	29.5	—	—
Choana width	—	—	42e
Choana length	—	—	29e
Skull table length, medial	—	96.1	133.9
Snout length, to posterior nares	412.1	—	—
Quadrate condyle width	—	43.2 (r)	56.3 (l)
Supratemporal fenestra width	—	70.9 (l)	86.4 (r)
Supratemporal fenestra length	—	66.6 (l)	75.5 (r)
Palatal fenestra length	—	96.5 (l)	119.1 (l)
Palatal fenestra width	42.0e	41.9e (l)	59.8
Pterygoid flanges width (across their lateral borders)	—	—	208.8
Incisive foramen length	21.1	—	—
Rostrum width at fourth maxillary alveoli	48.6	—	—
Rostrum width at notch for fourth mandibular tooth	37.7	37.3	—
Tooth row length	495.2e	—	—
Palatine bar width	33.0e	—	48.0e
Skull length	—	—	—
Skull height	—	70e	—

Measurements (mm) after Langston & Gasparini [8]. Measurements with missing data are omitted. Abbreviations:

e, estimate

l, left

r, right.

**Table 2:** Measurements of representative mandibles of *Gryposuchus pachakamue*.

	MUSM 987	MUSM1428
Mandible length	698.0e	—
Symphysis length	369.2	398.3
Tooth row length	414.5	479.3e
External fenestra length	49.0	—
External fenestra height	21.0	—
Glenoid fossa length	24.9	—
Retroarticular process length Mandible height at d4	85.7e	—
Mandible height	25.8	—
Mandible width at fourth tooth	49.4*	59.0*
Mandible width at the end of the symphysis	92.0*	102.2*
Splenial length in symphysis	133.9	145.0

Measurements (mm) modified from Langston & Gasparini [8]. Asterisk indicates doubling of measurement from one side.

Abbreviation:

e, estimate.

#### Skull

*Gryposuchus pachakamue* has a long and slender skull. The snout is parallel-sided and tubular in cross section. The anterior maxillary snout has slightly sinuate margins. Proportions of the rostrum correspond to those of *Gavialis gangeticus*: the rostral length/skull length index is 0.75 (0.76 in *G*. *gangeticus*) and the rostral width/postorbital width index is 0.27 (0.25 in *G*. *gangeticus*). In front of the orbits, the initial lateral expansion of the skull occurs at the level of the sixteenth maxillary alveolus and continues to widen gradually posteriorly. In dorsal view, the post-rostral skull outline is roughly triangular and the skull table is wide, trapezoidal in shape, and perforated by large supratemporal fenestrae. The supratemporal fenestrae are irregular in outline and widest posteriorly. The orbits are slightly wider than long and markedly smaller than the supratemporal fenestrae. The infratemporal fenestra is roughly ellipsoid in shape. The narial opening is heart-shaped, and its narial rim surface is interrupted anteriorly and anterolaterally by distinctive grooves, but no plateau-like shelf typical of adult males of *Gavialis* is observed in any specimen. The incisive foramen forms a slender, elongated isosceles triangle. The occipital plate is posteriorly inclined but appears to be at a lesser degree than in other South American gavialoids. Suborbital fenestrae are proportionally longer than those of *Gryposuchus colombianus* and *Gavialis gangeticus*, with the anterior end acute and the posterior margin broadly rounded. Pterygoid bullae are located laterodorsal to the posterior half of the palatine bridge.

**Fig 4:**
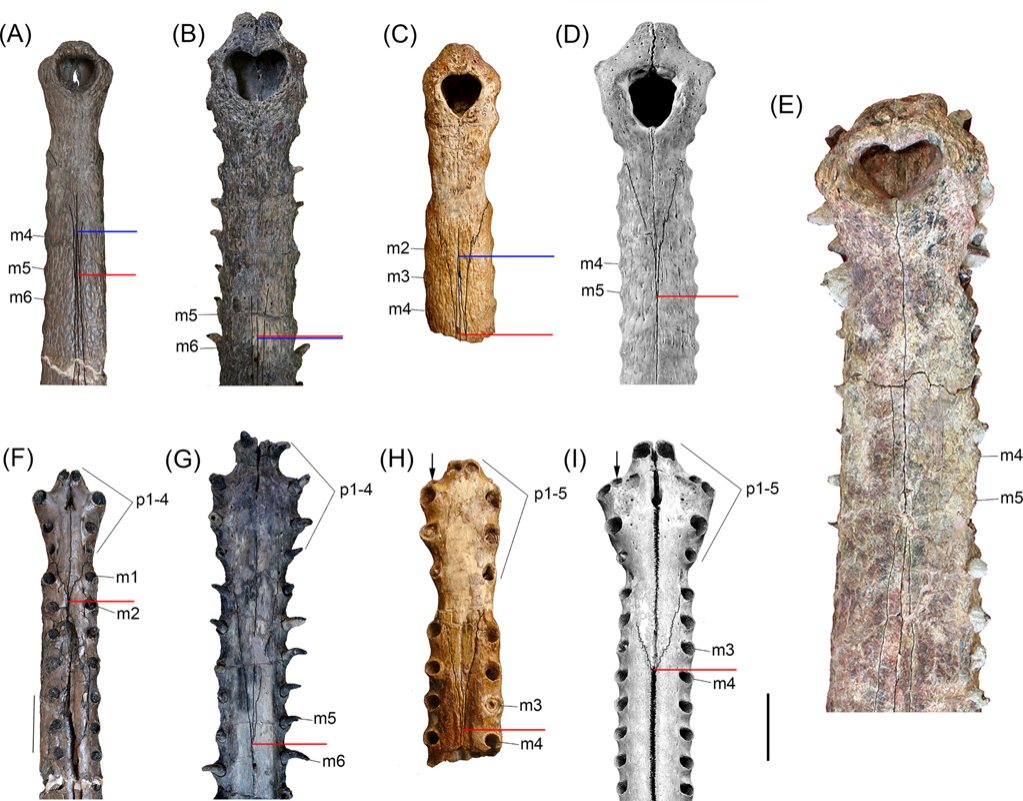
Comparisons between distal snouts of selected gavialoids. (A,F) *Gryposuchus pachakamue* in dorsal (A) and ventral (F) view (MUSM 1981). (B,G) *Piscogavialis jugaliperforatus* in dorsal (B) and ventral (G) view (SMNK 1282 PAL). (C,H) *Eogavialis africanus* in dorsal (C) and ventral (H) view (AMNH 5075). (D,I) *Gavialis gangeticus* in dorsal (D) and ventral (I) view (MNHN A5321). (E) *Gryposuchus colombianus* in dorsal view (IGM 184696). Red and blue horizontal bars indicate the posterior limit of the premaxillae and anterior limit of the nasals, respectively. Abbreviations: m2, m3, m4, m5, m6, maxillary tooth positions; p1-p4, p1-p5, premaxillary tooth series. Scale bar, 5 cm.

The premaxillae are expanded at the level of the anterior end of the external naris but not as much as in *Gavialis* or *Gryposuchus colombianus* (Fig 4). Long and slender posterior processes of the premaxillae reach the level of the fifth maxillary alveolus (Fig 4A). Thin anterior processes of the nasals are in extensive contact medially with the posterior processes of the premaxillae, a condition also observed in *Eogavialis africanus* (Fig 4C). Although the extension of these processes is not precisely symmetrical, their contact length roughly equals the narial opening length. As in all South American gavialoids, each premaxilla bears four alveoli and not five as in *Gavialis*, *Eogavialis*, and *Eothoracosaurus*, most probably by the loss of the second tooth loci of these latter taxa [5] (Fig 4H and 4I). In *Gr*. *pachakamue* and other South American gavialoids, where known, the first premaxillary alveoli are close one to another and separated by large gaps from the second alveoli. First and second tooth loci bear alveolar collars projecting ventrally relative to the palatal plane. Second alveoli are the biggest in the premaxilla whereas the fourth premaxillary alveoli are the smallest, as in *Gryposuchus* species [42] (Fig 4F). Relatively big foramina are located medial to the third and fourth premaxillary alveoli. The foramina are connected by shallow and bowed grooves. As in other *Gryposuchus* species, posterior ventral processes of the premaxillae are relatively short, reaching the level of the second maxillary alveoli. In *Piscogavialis*, they surpass the level of the fifth maxillary alveoli (Fig 4G). Ventrally, the premaxilla bears two anterior medial processes that project into the incisive foramen, and the premaxillary-maxillary suture is jagged and not linear as in all other gavialoids.

The maxillae are long and tubular resembling those of *Eogavialis*, and not dorsoventrally flattened as in *Gryposuchus colombianus* and *Piscogavialis*. Dorsally, the maxillae are not in contact due to the forward projecting nasals that contact the slender posterior processes of the premaxillae. As in other *Gryposuchus* species, modest alveolar salients are observed along most of the maxillary margins. Each maxilla bears 22 tooth positions, all of them subequal in size, exclusive of the last two maxillary alveoli that are the only significantly smaller alveoli (Fig 2B). Ventrally, the intermaxillary suture extends posteriorly until the level of the fifteenth tooth position. The edge of the maxillary tooth alveoli is higher than the palatal space between both tooth rows. The palatal surface is relatively convex. The anterior interalveolar length is notably larger than the diameter of adjacent alveoli. This length decreases progressively posteriorly, to become equal to alveoli diameter at the fifteenth tooth position and even smaller posterior to it. Dorsally, at the level of the thirteenth tooth position, the maxillae contact the anterior extension of the lacrimals. There is no posterior process of the maxilla between the lacrimal and jugal, in contrast to the condition in *Gryposuchus colombianus*. The maxillae have a long edentulous posterior process that reaches the level of the postorbital bar (e.g., MUSM 900). Maxillae have no contact with the prefrontals.

The nasals are extremely long, slender bones with close and extensive anterolateral contact with the premaxillae. Additionally, the nasals contact the maxillae and lacrimals laterally, the prefrontals posteriorly, and the frontal posteromedially. The nasals are in contact with each other along the sagittal axis for most of their length. Posteriorly, they are separated by the pointed anterior process of the frontal, almost at the level of the anterior extension of the jugals. The lateral margins of the nasals slightly diverge posteriorly, reaching their greatest transverse diameter just ahead of the anterior processes of the prefrontals. The posterior end of the nasals reach the level of the anterior margins of the orbits, resembling in this aspect *Eogavialis* and *Gryposuchus colombianus*, and in distinction from *Piscogavialis* and *Ikanogavialis* in which the posterior end of the nasals is located far anterior to this position. The condition in *Gavialis* is intermediate in this aspect. Whereas the posterior process of the nasal is pointed in all South American gavialoids where this area is known, including *Gryposuchus pachakamue*, in *Gavialis* it is strongly denticulate.

The lacrimals are large and roughly triangular in shape. They contact the maxillae and jugals laterally and the nasals and prefrontals medially. Posteriorly, they form the anterior margin of the orbits. The anterior process of the lacrimal exceeds in length that of both the frontal and prefrontal. A small, discrete knob occurs at the orbital margin, lateral to the prefrontal-lacrimal suture.

The jugals form the ventral margins of the orbits and infratemporal fenestrae. In *Gryposuchus pachakamue* this bone differs from other *Gryposuchus* species and most other gavialoids, and it more closely resembles the general pattern observed in non-gavialoid crocodylians. Gavialoids, such as *Gryposuchus colombianus*, *Gr*. *croizati*, *Ikanogavialis*, *Gavialis*, and to a lesser degree *Siquisiquesuchus*, present a deep notch immediately anterior to the postorbital pillar. However, in non-gavialoid crocodylians as well as some gavialoids such as *Gryposuchus pachakamue*, *Eothoracosaurus*, *Eosuchus*, and *Piscogavialis*, the jugal orbital rim progressively descends lateral to the postorbital pillar, thus the ventral margin of the orbit is gently circular. Additionally, in these latter gavialoid taxa and in *Siquisiquesuchus*, the postorbital pillar reaches the horizontal bar of the jugal medially and a longitudinal sulcus is present between these structures. This latter condition also differs from the distinctive feature observed in gavialoids such as *Gavialis* and *Gryposuchus* species other than *Gr*. *pachakamue*, in which the postorbital bar lies flush with the lateral surface of the jugal.

The prefrontals are short and rhomboid in shape. They are separated from each other by the frontal and nasals and form part of the anterodorsal orbital margin. From the orbit, the prefrontal-frontal suture follows a gentle semicircular path. In contrast, in *Gavialis* and *Gryposuchus colombianus* this suture is sharply angulated, a condition associated with their possession of fully “telescoped orbits” (see below).

The frontal bears a long anterior process that largely exceeds the anterior end of the prefrontal and that of the orbit too. Posteriorly, the main surface of the frontal is slightly concave and weakly sculptured (Fig 2A). Laterally, along the fronto-postorbital suture the skull table is markedly higher than the surrounding areas. Frontal participation in the orbital rim is limited to the posteromedial corner. The frontal in the skull table is anteroposteriorly short due to the large size of the supratemporal fenestrae. Frontal sutures on the skull table are well preserved in MUSM 1681 and MUSM 2032 (Fig 3A and 3B). The fronto-parietal suture is markedly concavoconvex (i.e., M-shaped) between the supratemporal fenestrae and is linear laterally. It runs along the anterior border of, and only briefly enters into, the supratemporal fenestra as in *Gryposuchus colombianus*. In MUSM 900 the suture runs along the dorsal surface of the skull table, just grazing the margin of the fenestra. The frontal bone and skull table are not elevated relative to the rostrum. The skull table presents an uneven surface, being depressed between the orbits but higher along the postorbital-frontal suture and parietal-supraoccipital region.

The postorbital bones on the skull table are relatively flat and lightly built. They form the anterior corner of the skull table and lack the anterolateral postorbital process characteristic of *Gavialis*, *Gryposuchus colombianus*, *and Gr*. *neogaeus*. The development of this anterolateral process is variable in *Gryposuchus croizati* and is small in *Siquisiquesuchus* and *Piscogavialis*. The postorbital medially contacts the frontal and slightly contacts the parietal, at the rim of the supratemporal fenestra. The postorbital bone forms part of the dorsal portion of the postorbital bar. As in other gavialoids, the postorbital bar is robust and longer than wide in cross section. On the lateral side of the postorbital bar, lying entirely on the postorbital bone, there is an anteroposteriorly-oriented, crest-like bump, similar to that of *Gryposuchus colombianus* and *Piscogavialis*. In MUSM 900, the largest specimen, the bump presents an anterolateral spine similar to that of *Gavialis* [43]. The suture of the postorbital bone with the jugal is located lower on the postorbital bar, although its precise pattern is not clearly preserved. The lowermost descending process of the postorbital is located posteriorly; it reaches the level of the horizontal bar of the jugal, and contacts the ectopterygoid medially. The postorbital bar is inset from the anterolateral margin of the skull table. Beneath this margin, on the excavated lateral surface, most specimens possess two big foraminae although their size, number, and positions are variable across specimens. It is impossible to determine if the postorbital contacts the quadratojugal medially.

The squamosals are incomplete in the holotype but well preserved in MUSM 1681, MUSM 2032, and particularly in MUSM 900; they occupy the posterolateral corner of the skull table in each. *Gryposuchus pachakamue* exhibits prong-like posterior extensions of the squamosal, but they are comparatively shorter than those of most South American gavialoids and *Argochampsa* (Fig 1E) [6, 9,10]. In *Gryposuchus colombianus*, this region is not well preserved, but it seems that long “prongs” were present as well [8]. On the skull table, the bar that posteriorly limits the supratemporal fenestrae is relatively thin (MUSM 1681), similar to that of *Gavialis* and *Gryposuchus colombianus*, but to a lesser degree than in *Piscogavialis* and *Gr*. *neogaeus* [44,45]; in *Eogavialis*, this bar is comparatively wider. The anterior process of the squamosal lateral to the skull table runs anteroventrally, reaching the posterodorsal end of the postorbital pillar.

The parietal forms the medial and posteromedial margins of the supratemporal fenestrae. Anteriorly, the parietal surface between the supratemporal fenestrae is horizontal. The posterior surface of the parietal gently ascends, thus its main body slopes anteriorly and also laterally, as is typical in gavialoids. Posteriorly, it contacts the supraoccipital along the sagittal axis. The parietal interfenestral bar is comparatively thinner than that in *Gavialis* and *Siquisiquesuchus* [10].

The supraoccipital forms an inverse triangle in the occipital plate, excluded from the foramen magnum. It also bears a small, rhomboid-shaped, dorsomedial projection on the cranial roof that points posteriorly. Together with the parietal, this posterior extension becomes a prominent vertical medial crest on the occipital plate (observed in MUSM 1681). The postemporal fenestrae are hardly discernable in any of the specimens, and they seem to be small. Available specimens show asymmetrical development of the postoccipital processes (processus postoccipitales of Kälin) [46] and surrounding area of the supraoccipital, although the so-called “nuchal crest” is not hypertrophied as it is in *Gryposuchus colombianus* [8].

The quadratojugal forms the posterior margin and corner of the infratemporal fenestra. The anterior process of the quadratojugal comprises a robust spine, as is usually observed in gavialoids. Dorsomedially, the ascending process that bounds most of the posterior margins of the infratemporal fenestra is very thin and long; its dorsal end is not discernible. Sutural contacts of the quadratojugal with the jugal and the quadrate are not parallel, in contrast with the condition in *Piscogavialis*.

The quadrates are robust and short. The quadrate ventrally bounds the otic aperture and, although incomplete, it seems to occupy part of its posterior margin; thus the quadrate-squamosal suture might have reached the otic aperture along the posterior wall. The small foramen aëreum is located on the mediodorsal surface of the quadrate. The axis of the mandibular condyles is oblique, as it generally is in gavialoids. Medial and lateral condyles are clearly discernable, with the medial one deflected ventrally as in South American gharials, where known, such as in *Piscogavialis*, *Siquisiquesuchus*, and *Gryposuchus croizati*.

The laterosphenoids are partially preserved in the holotype, as well as in MUSM 2032 and MUSM 1681. The anterior dorsal margin of the laterosphenoid, as well as the capitate process, is oriented lateromedially. The dorsal capitate process is massive, and its whole anterior portion is relatively flat.

**Fig 5:**
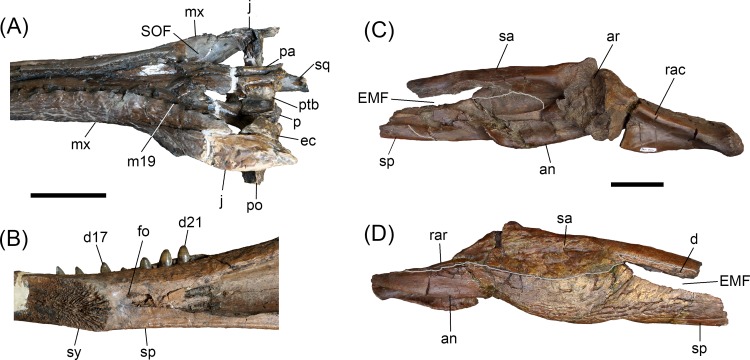
Noteworthy anatomical features of *Gryposuchus pachakamue*. (A) Suborbital fenestra region of the holotype skull (MUSM 1981) in lateroventral view showing the pterygoid bullae (ptb). (B) Post-symphyseal mandible (MUSM 987) in posteromedial view showing a foramen (fo), that probably is the aperture of the foramen intermandibularis oralis. (C,D) Posterior portion of the right mandible (MUSM 1440) in dorsomedial (C) and lateral (D) view. Abbreviations: an, angular; ar, articular; d17, d21, dentary tooth positions; ec, ectopterygoid; EMF, external mandibular fenestra; fo, foramen; SOF, suborbital fenestra; j, jugal, mx, maxilla; p, parietal; pa, palatines; po, postorbital; ptb, pterygoyd bullae; rac, retroarticular crest; rar, retroarticular process of the mandible; sa, surangular; sp, splenial; sq, squamosal; sy, symphysis. Scale bars, 5 cm.

The palatine bones cover the entire ventral surface of the bridge between the suborbital fenestrae. They are roughly parallel-sided and transversely convex along this bridge. From the anterior limit of the suborbital fenestrae, the lateral margins of the palatine bones converge to a point approximately at the level of the fifteenth maxillary alveolus. The anterior limit of the suborbital fenestra is located at the level of the nineteenth maxillary alveolus (Fig 5A). The palatine-pterygoid suture lies anterior to the rear margin of the suborbital fenestra.

The ectopterygoids are incomplete and badly damaged, but preserved regions are informative. The anterior process of the ectopterygoid is thin and long, resembling that of *Piscogavialis*, and comprises the posterior half of the margin of the suborbital fenestra. The anterior tip runs medially to the maxillary tooth row until the level of the anterior limit of the penultimate alveolus, as in *Piscogavialis*. The distance between the tooth row and the anterior process of the ectopterygoid is less than in *Gavialis*. In ventral view, *Gryposuchus pachakamue* and *Piscogavialis* display a substantial contact between the ectopterygoid and jugal, whereas this contact is smaller in the extant gharial *Gavialis gangeticus*. Available evidence from the same anatomical area is not clear in other South American fossil gharials. *Eogavialis* specimens do not preserve details of this region, other than in exhibiting a large ectopterygoid-jugal contact. Differing from all other gavialoids, in *Argochampsa* the ectopterygoid stops far behind the tooth row [47]. The posterior process of the ectopterygoid is short in *Gryposuchus pachakamue*; thus contact with the pterygoid flanges is reduced relative to the condition in other gavialoids.

The pterygoid widely separates the ectopterygoids from the palatines. In the holotype of *Gryposuchus pachakamue*, pterygoid bullae in intimate contact with the palatines are observed dorsal to the posterior portion of the palatal bridge. The bullae are neither ovoid in shape nor smooth-surfaced, as in other gavialoids; instead they are kidney-like in shape with irregular prominences and depressions. They are relatively small, flat, and project slightly laterally into the suborbital fenestrae, as in the type specimen of *Gryposuchus colombianus*. The choana is distorted in MUSM 900 but is not preserved at all in any other specimen.

The exoccipitals are best preserved in MUSM 1681. As in most gavialoids, they are visible in dorsal view, but not as much as in *Piscogavialis*, *Gryposuchus colombianus*, and *Gr*. *croizati*. The paraoccipital processes are long and encompass a ventral plate-like expansion that completely covers the cranioquadrate foramen. Dorsal to the foramen magnum, the exoccipitals and the supraoccipital are collapsed by deformation, thus positional relationships of these bones are obscured. The ventral processes of the exoccipitals laterally embrace the basioccipital tubera. These processes are robust and anteroposteriorly extended.

The basioccipital is typically gavialoid in being low and laterally expanded ventral to the condyle, producing two pendulous tuberae. The bassioccipital plate is smooth, exclusive of the lateral and ventral margins, in which tuberosities are well developed. Medially, the ventral margin of the plate is excavated. The basisphenoid is anteroposteriorly broad anterior to the basioccipital. The medial eustachian foramen is wider than long.

The braincase region is badly damaged around the prootic in all of the specimens.

#### Mandible

The mandibular rami (MUSM 987; Fig 1D) are sutured anteriorly, through a long rostral symphysis, to form a Y-shaped structure. Although long, the symphyseal region is proportionally shorter than in *Gavialis*, *Ikanogavialis*, *Siquisiquesuchus*, and *Piscogavialis*. The mandible is low along the symphysis; and its height progressively increases posteriorly until the level of the glenoid fossa. The external mandibular fenestra is small, eye-shaped and occurs comparatively closer to the articular region than in *Gavialis*.

The dentary bone is long, transversely expanded at the level of the second alveolus and restricted to the lateral wall of the mandibular ramus between the end of the tooth series and the external mandibular fenestra. Posteriorly, the dentary reaches the rear margin of the external mandibular fenestra (EMF). The first 15 alveoli are implanted within salients along the lateral border of the dentary, providing a sinuous profile to this margin. The dentary (MUSM 987) bears 22 alveoli, all of them sub-equal in size, exclusive of the first and fourth, which are of greater diameter: the second and third are the smallest of the whole series. *Gryposuchus colombianus* and *Gr*. *croizati* present similar alveolar counts (i.e., 22–23) and the same general dental pattern described above. Differences in *Gr*. *pachakamue* relative to these other *Gryposuchus* species include: proportionally higher and more tubular dentary, stronger alveolar salients, and no constriction between the fourth and fifth alveoli (this last character only pertains to comparisons with *Gr*. *colombianus*). There are at least four tooth positions located posterior to the level of the mandibular symphysis, as in *Piscogavialis*, whereas other gavialoids generally possess no more than three. These alveoli are located along the medial limit of the dentary and their lingual wall is formed either by the splenial (i.e., nineteenth and twentieth alveoli) or the surangular (i.e., twenty first and twenty second alveoli).

**Fig 6:**
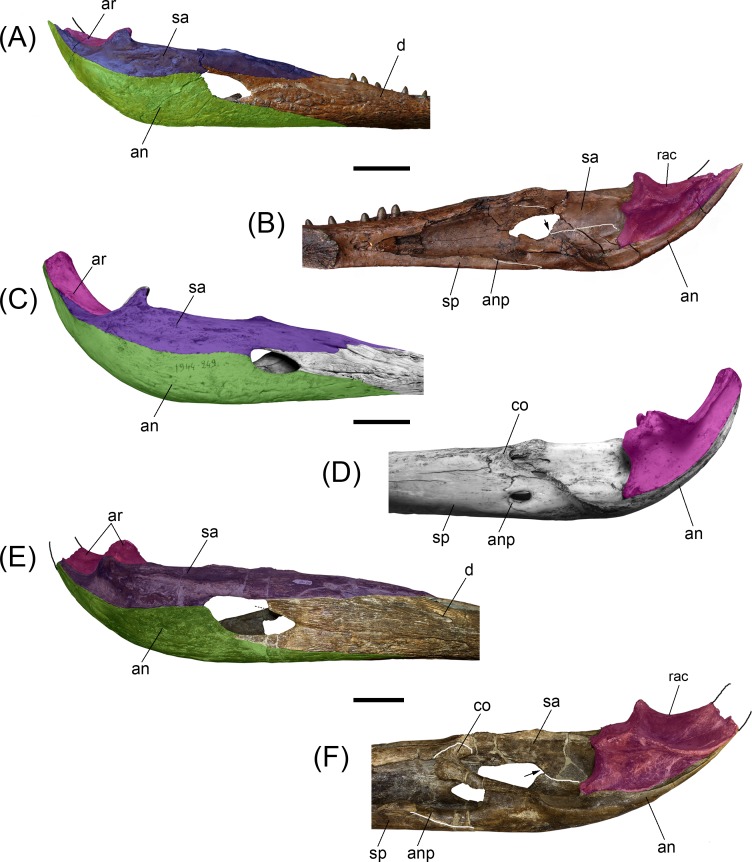
Comparisons between post-symphyseal mandibles of selected gavialoids. (A,B) *Gryposuchus pachakamue* (MUSM 987) in lateral (A) and medial (B) view. (C,D) *Gavialis gangeticus* (MNHN A-5312) in lateral (C) and medial (D) view. (E,F) *Piscogavialis jugaliperforatus* (MUSM 449) in lateral (E) and medial (F) view. The arrows in (B) and (F) show the ventral process of the surangular in medial view. Abbreviations: an, angular (green color in lateral views); anp, angular process; ar, articular (fuchsia color); co, coronoid; d, dentary; rac, retroarticular crest; sa, surangular (purple color in lateral views); sp, splenial. Scale bars, 5 cm.

The splenials wedge out between the dentaries at the level of the twelfth dentary alveolus. As also present in South American gavialoids for which the splenial is known, the anterior process is long, slender, and constricted between the dentaries along the symphysis. Its lateral margin is bowed medially. In MUSM 1428, a right mandible, the medial surface of the splenial, situated within the symphysis, shows no perforation of the foramen intermandibularis oralis. Absent in *Tomistoma schlegeli*, this foramen occurs within the symphysis of *Gavialis gangeticus* at the level of the twenty-second alveolus [43]. We identified a distinctive foramen in *Gryposuchus pachakamue* (MUSM 987 and MUSM1428) and *Eogavialis africanus* (AMNH 5069)–probably homologous to that of *Gavialis*–situated just behind the symphysis, within the medially divergent walls of the splenial, at the level of the twentieth alveolus (Fig 5B). Posterior to the symphysis, the splenial occupies the internal half of the mandibular rami. The splenial is excluded from the margins of the foramen mandibularis caudalis, whereas its ventral posterior process extends beyond the rear limit of this foramen (Fig 6B). Other details of the splenial anatomy, particularly the positional relationship to the angular or coronoid, are not preserved in any specimen.

The surangular extends from the medial anterior border of the twentieth alveolus to the lateral surface of the retroarticular process. Within this process, the surangular is partially preserved in MUSM 987, but fully preserved in MUSM1440 (Fig 5D). This latter specimen shows that the surangular fails to reach the posterior tip of the retroarticular process. In lateral view the surangular is low. It is restricted to the posterior angle of the EMF due to the posterior expansion of the dentary and remarkable depth of the angular that embraces this fenestra posteriorly. This condition probably also pertains to *Piscogavialis* (Fig 6C) and *Gryposuchus colombianus* [8]. Externally, at the level of the glenoid fossa, there is a large anterodorsally facing foramen (Fig 2D). Among gavialoids, a similar foramen is observed only in *Eogavialis africanus* (SMNS 11785). Just behind this foramen, the surangular covers the postglenoid process of the articular; therefore this process is hardly seen in lateral view as in *Gavialis* (Fig 6A and 6C) but in contrast to *Piscogavialis* in which the surangular ascending lamina is relatively small (Fig 6E). Medially, the surangular almost reaches the lower margin of the EMF as observed in most crocodylians and South American gharials [8,44], except for *Piscogavialis* (Fig 6E). Posteriorly, the surangular-angular suture reaches the articular at its ventral tip. Proportions of the foramen mandibularis caudalis are similar in *Gryposuchus pachakamue* and *Piscogavialis*, with this foramen being relatively longer than in *Gavialis*.

The angular is excluded from the ventral margin of the EMF being only restricted to its posterior margin, a feature also observed in the newly recovered material of *Piscogavialis* (MUSM 449; Fig 6E). Other gavialoids such as *Gavialis*, *Gryposuchus neogaeus*, and *Gr*. *colombianus* [8,44] typically possess a small posterior process of the dentary; therefore the angular borders most of the EMF ventrally. The angular dorsally approaches the posterior process of the dentary, but fails to contact it. An angular-dentary contact in this position is described for *Gr*. *colombianus*, but to our knowledge this area is damaged in all referred specimens of that species and this condition is best considered to be unknown in *Gr*. *colombianus*. The angular forms the ventral section of the retroarticular process in *Gryposuchus pachakamue*. The posterior tip of the angular is not preserved.

The articular bone overlies the angular. It is posteriorly and medially inclined relative to the condition in *Gavialis* (Fig 6). The articular also is inclined in *Piscogavialis*, a condition probably correlated with the ventromedial projection of the medial hemicondyle of the quadrate. As a consequence, the medial fossa of the retroarticular dorsal surface can be observed in medial view. A prominent longitudinal crest on the dorsal surface of the retroarticular process limits this fossa laterally. This crest is also well developed in *Piscogavialis*, and other South American gharials, such as *Gryposuchus colombianus*, and *Siquisiquesuchus* [10]. Within the glenoid fossa, the articular-surangular suture runs diagonally from its concave anterior margin to the lateral limit of the postglenoid crest.

#### Dentition

The adult upper dentition formula is 4 premaxillary + 22 maxillary teeth. This count is similar to other *Gryposuchus* species [8,9] but much less than that of *Piscogavialis* [45] and *Ikanogavialis* [48]. The number of tooth loci in the lower jaw is 22.

Tooth crowns are conical in shape and inclined posterolingually in the plane formed by the fore and aft carinae. Beyond this general morphological similarity, proportions vary substantially relative to tooth locus position in both the upper and lower jaws. Although anterior teeth are long and slender, they do not show the sigmoid-shaped crown of other gavialoids. Posterior teeth are short, robust, and slightly blunt. Although weak longitudinal striae are observed, the surface can be described as virtually smooth.

#### Juvenile specimens

Iquitos gavialoid specimens of relatively smaller sizes are referred here as juvenile individuals of *Gryposuchus pachakamue*. MUSM 1988 is an incomplete skull lacking the snout (Fig 3C, 3G and 3H). The width of the skull across the postorbital bars is 90.5 mm whereas in the holotype this diameter is around 178.0 mm. This specimen preserves the skull table as well as the orbital and occipital regions. The medial portion of the posterior region of the skull table has been compressed, thus the foramen magnum is collapsed and the occipital plate is partially distorted. Compared with specimens described above as possessing the adult morphology of *Gryposuchus pachakamue*, the juvenile MUSM 1988 has a slender postrostral skull, due to the lesser degree of posterior divergence of the jugal lateral margins. The orbits are elongated and equivalent in size to the supratemporal fenestrae. In contrast to the juvenile condition, the adult morphology shows short, wide orbits, and large supratemporal fenestrae, suggesting that a marked allometric development involving the orbital and postorbital regions occurs during ontogeny [16]. Juvenile fenestral shape and proportions resemble those of the holotype of *Piscogavialis jugaliperforatus*, the latter clearly representing an adult individual. The fronto-parietal suture lies entirely on the skull table without contacting the margin of the supratemporal fenestra. The posterior process of the nasals surpasses the anterior margin of the orbits. The interorbital bridge width is equivalent to the orbit transverse diameter, as in adult individuals. The basioccipital plate and tuberae are well preserved in MUSM 1988 (Fig 3H). The basioccipital plate is wider and dorsoventrally shorter than in the adult condition represented by MUSM 1681 (Fig 1E). The foramen carotideum posterior is well exposed in posterior view, next to the lateral margin of the exoccipital ventral processes. These processes are robust and reach the tuberae. The tuberae are separated medially by a depression posterior to the medial Eustachian foramen. The lateral margins of the basioccipital plate are parallel and composed entirely by the exoccipitals. In adult specimens (Fig 1E), lateral margins become divergent ventrally and a significant portion of the tuberae are extended ventral to the exoccipital, yielding a pendulous shape for the basioccipital-exoccipital structure.

MUSM 1727 comprises a partial snout preserved until the level of the ninth maxillary alveolus (Fig 3K and 3L). As in MUSM 1988, this juvenile snout is comparatively more slender than in specimens representing the adult morphology. Major differences between juveniles and adults lie in the relatively smaller size of the alveoli and, consequently the larger diastemata between adjacent tooth loci, within the juvenile specimens in both upper and lower dental quadrants (Fig 3L, 3N and 3O). Partial mandibles comprising the symphyseal region have been recovered from Localities IQ 136 (MUSM 1682: MZ5; Fig 6O) and IQ 26 (MUSM 1439: MZ8; Fig 6N). We estimate the time span separating these localities in around 3 million years. These represent animals of equivalent size, although smaller alveoli in the specimen recovered from the younger deposits indicate that it probably represents an earlier ontogenetic stage and would have had a larger adult size. Other features of the juvenile specimens, such as the extension of the splenial symphysis and the postsymphyseal tooth loci, seem to be consistent with those of adult individuals.

#### *Gryposuchus* cf. *pachakamue* from Urumaco

Other than differences in size, the anatomical traits of AMU CURS 12, a partial skull from the Late Miocene Urumaco Formation of Venezuela [34] are essentially identical to those of the new species *Gryposuchus pachakamue* (Fig 3D). As in the Peruvian specimens, AMU CURS 12 bears a trapezoidal skull table that is extensively perforated by supratemporal fenestrae, and circular and only moderately “telescoped” orbits. Additionally, the Venezuelan specimen resembles *Gr*. *pachakamue* and also differs from other *Gryposuchus* species in having its interorbital bridge width equivalent to the transverse diameter of the orbit, and a postorbital pillar contacting the horizontal bar of the jugal medially. Bone sutures are not discernable on the Venezuelan specimen.

### Anatomy of the orbital region in gavialoids

**Fig 7:**
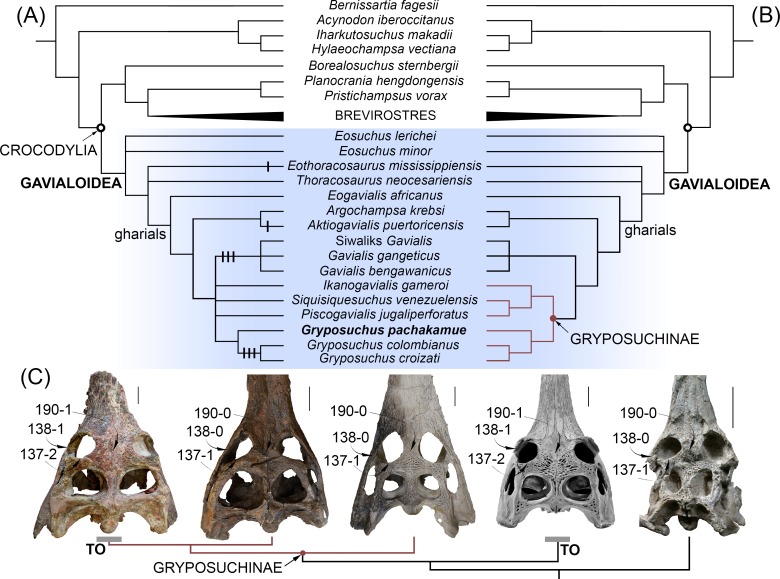
Phylogenetic position of *Gryposuchus pachakamue* within crocodylians. (A) Strict consensus cladogram of 45 most parsimonious trees based on parsimony analysis of the complete data matrix (S1 Appendix). Apomorphic character states associated with a “telescoped” orbit condition are plotted on the cladogram as black lines (i.e., 137–2, 138–1, and 190–1). (B) Strict consensus cladogram of 24 optimal trees in a second parsimony analysis performed after removing character state 137–2 and character 138 from the data matrix (S2 Fig). (C) Parallel acquisition of a fully “telescoped” orbit condition (TO) in advanced South American *Gryposuchus* and Indian *Gavialis*. Selected character states of the circumorbital region are indicated with arrows. From left to right: *Gryposuchus colombianus* (IGM 184696), *Gryposuchus pachakamue* (MUSM 900), *Piscogavialis jugaliperforatus* (SMNK 1282 PAL), *Gavialis gangeticus* (MNHN A5321), and *Argochampsa krebsi* (OCP DEK-GE 333). Scale bars, 5 cm.

Circumorbital bones include the frontal, prefrontals, lacrimals, jugals, and postorbitals. The configuration of these bones determines the orbital shape and the general position of the eyeballs in crocodylians. In the adult individuals of *Gryposuchus pachakamue*, the orbits are slightly wider than long, closer in shape to those of *Eogavialis africanus* than to the circular orbits of *Gavialis* and other *Gryposuchus* species. In contrast to the adult condition, the orbital shape and proportions of a juvenile specimen (MUSM 1988; Fig 3C) resemble those of most crocodylians, including gavialoids such as *Piscogavialis* and *Eothoracosaurus*, in which the orbits are comparatively longer anteroposteriorly. The relative diameter of the interorbital bridge (i.e., frontal bone width/orbital width ratio) defines the separation between the orbits. Brevirostrine crocodylians, *Eogavialis*, *Piscogavialis*, and *Ikanogavialis* generally bear a narrow interorbital bridge diameter (i.e., ratio <1.0) compared to *Gryposuchus pachakamue*, in which the width of the frontal bone is essentially equivalent to the width of the orbit and this ratio is consistently higher (more slender interorbital bridge) than that of adult *Gavialis* and other species of *Gryposuchus* (i.e., ratio >1; Fig 3 and Fig 7C). The dorsal orbital margins are upturned in many alligatoroids and crocodyloids [13,16]. Among gavialoids, *Eogavialis*, *Gryposuchus pachakamue*, *Piscogavialis*, and *Siquisiquesuchus* have also upturned dorsal orbital margins, but to a lesser degree than in *Ikanogavialis*, *Gavialis*, and other *Gryposuchus* species. In these latter taxa, dorsal and posterior margins are sharp and projected towards the orbits. Similarly, the anterior orbital margin (i.e., lacrimal bones) of *Eogavialis*, *Gr*. *pachakamue*, and *Piscogavialis* lies flush with the rostral surface contrasting with the raised anterior margins of other gavialoids. The jugals of *Gr*. *pachakamue* differ from those of other *Gryposuchus* species and most other gavialoids, more closely resembling the general pattern of non-gavialoid crocodylians. Gavialoids such as *Gryposuchus colombianus*, *Gr*. *croizati*, *Ikanogavialis*, *Gavialis*, and to a lesser degree *Siquisiquesuchus*, present a deep notch immediately anterior to the postorbital pillar. However, in non-gavialoid crocodylians and some gavialoids such as *Gryposuchus pachakamue*, *Eothoracosaurus*, *Eosuchus*, and *Piscogavialis*, the jugal orbital rim progressively descends lateral to the postorbital pillar, thus in those taxa the ventral margin of the orbit is gently circular. Additionally, in those taxa as well as in *Siquisiquesuchus*, the postorbital pillar reaches the horizontal bar of the jugal medially and a longitudinal sulcus is present between these structures. This latter condition also differs from a distinctive feature observed in many other gavialoids, such as *Gavialis* and other *Gryposuchus* species, in which the postorbital bar lies flush with the lateral surface of the jugal (Fig 7C). Postorbital bones on the skull table are relatively flat in *Gr*. *pachakamue*. They form the anterior corner of the skull table and lack the anterolateral postorbital process characteristic of *Gavialis*, *Gryposuchus colombianus*, and *Gr*. *neogaeus*.

In summary, in contrast to the relatively conservative morphology of the orbital region of most brevirostrine crocodylians, all gavialoids present conspicuous modifications within the circumorbital anatomy associated with possessing protruding eyes or “telescoped” orbits. The degree of development of the “telescoped” orbit condition varies among gavialoid taxa and usually includes at least some of the following character states: upturned anterior, dorsal, and posterior orbital margins; anterolateral postorbital process; ventral orbital margin with a prominent notch; postorbital pillar laterally displaced; and orbits widely separated. Adult *Gavialis*, *Gryposuchus colombianus*, and *Gr*. *croizati* possess all of these traits and therefore the fully “telescoped” orbit condition. Juvenile individuals of *Gavialis* lack widely separated orbits; other characters associated with the “telescoped” orbits condition are recognized but they are less marked than in adult skulls. The general configuration of this area in the new gavialoid *Gryposuchus pachakamue* depicts weak development of “telescoped” orbits. This is interpreted as an incipient condition for more extensive telescoping of the orbits in later diverging relatives, based on ancestral state transformations determined from the maximum parsimony phylogeny.

### Phylogenetic analysis

Our first parsimony analysis retained 45 equally optimal trees with a minimum length of 538 steps. The strict consensus phylogeny (Fig 7A) calculated from those trees yielded the following statistics: length = 553; consistency index (CI) = 0.461; retention index (RI) = 0.743. Our strict consensus tree shows general coincidence with previous morphological and molecular analyses for major relationships within crocodylian clades [5,6,13], but as in other morphological analyses, it differs markedly from results based only on molecular data in the hypothesized affinities between *Gavialis* and *Tomistoma* (see Results in [3,4]). Thus, we found strong Bremer support for the monophyly of Gavialoidea, with *Gavialis* having a much closer relationship to Cretaceous gavialoid taxa such as *Eothoracosaurus* than to extant *Tomistoma*, the latter being most closely allied with *Crocodylus* within the Crocodyloidea. Within the Alligatoroidea, *Culebrasuchus mesoamericanus* is closer to *Alligator mississippiensis* than to *Caiman crocodilus* [11].

The new Pebasian species, *Gryposuchus pachakamue*, is recovered within gavialoids as the sister taxon of the *Gr*. *colombianus* + *Gr*. *croizati* clade. Thus, our results suggest that all known Amazonian gavialoids belong to a single monophyletic taxon (the inclusive species of *Gryposuchus*) characterized by association with riverine and lacustrine-tidal paleoenvironments. On the other hand, relationships among South American taxa usually associated with coastal marine paleoenvironments show no resolution: *Siquisiquesuchus*, *Piscogavialis*, and *Ikanogavialis* lie in a polytomy together with Amazonian *Gryposuchus* and Indo-Asian *Gavialis*. As a consequence, this first analysis finds no support for the monophyly of a clade comprising South American gavialoids, as had been suggested by previous studies [10,39,49]. This clade, namely Gryposuchinae as proposed by Vélez-Juarbe et al. [39], was usually linked only with weak support or collapsed when the analysis included *Argochampsa* [9]. It is noteworthy that we found support, although low, for a novel association between African *Argochampsa* and the Caribbean taxon *Aktiogavialis*. This clade is supported by the presence of long supratemporal fenestrae (character 191–0), a character state that implies a reversal within these gavialoids. This might be considered problematic since *Argochampsa*, as an early representative of the clade, could have retained the ancestral condition for Crocodylia if character polarization based on the phylogenetic position of “thoracosaurs” is erroneous. Nevertheless, further comparisons between *Argochampsa* and *Aktiogavialis* allows us to recognize other similarities not yet included in phylogenetic analyses, but potentially supporting their close relationship, such as the relative proportions of the skull table in dorsal view and the presence of a distinctive shallow fossa in the anterior margin of the supratemporal fenestra. This fossa has not been identified in any other crocodylian species at any ontogenetic stage [39], but it is clearly visible in the holotype of both *Argochampsa* and *Aktiogavialis*, although barely discernable in other individuals of the African taxon *Argochampsa* (Stéphane Jouve, pers. comm., 2015). The *Argochampsa* + *Aktiogavialis* group is found as most closely related to the clade of South American gavialoids plus Indo-Asian *Gavialis*, and with *Eogavialis* as the nearest outgroup to all of these taxa. Therefore, African *Argochampsa* is more closely related to Indo-Asian *Gavialis* as was previously suggested by others [6,9], than it is to African *Eogavialis*. In fact, both Paleogene African taxa, *Argochampsa* and *Eogavialis*, share key characters with more anatomically-derived gavialoids like *Gavialis*. The clade encompassing this subset of late-diverging gavialoids is here termed “gharials” (Fig 7) and it is characterized by a posteriorly pointing supraoccipital (character 160–1), anteroposteriorly wide basisphenoid (character 172–1), robust exoccipital ventral process to basioccipital tubera (character 176–1), and basioccipital plate with ventrally divergent sides (character 196–1). Other conspicuous features of the gavialoid temporal region present in *Gryposuchus pachakamue*, such as ovoid infratemporal fenestra (character 204–1) and anteriorly flaring squamosal groove (character 147–1), also are observed in the tomistomine *Thecachampsa americana* and might represent independent, convergent acquisitions. As in most crocodylians, the infratemporal fenestra is triangular in *Gavialis* (character 147–0), and is most parsimoniously regarded as a reversal in this latter taxon. Paleocene *Eosuchus* and Cretaceous “thoracosaurs” from the northern hemisphere are identified in this analysis as the first and second basalmost diverging branches among the gavialoids, respectively.

To better understand morphological transformations, we performed a second analysis, this time excluding individual characters revealed in the first analysis as highly homoplastic, since results suggested that they were acquired independently up to three times among later-diverging gavialoids, including *Gryposuchus* and *Gavialis*. In addition, this analysis collapsed states of character 138 (as they may not be independent) and deleted state 2 from character 137, both of which describe morphology of the orbital margin associated with “telescoped” orbits, which the all-data parsimony analysis indicate clearly evolved multiple times independently within gavialoids (see Discussion). Regarding character 138, two states were combined in this second analysis as all taxa with state 2 also preserve state 1 (non-independent states), and all taxa previously coded as having state 2 (i.e., 138–2: dorsal and posterior orbital edges upturned) were recoded as 1 (i.e., 138–1: dorsal edges of orbits upturned). This search retained 24 equally optimal trees, with a length of 533 steps. The corresponding strict consensus tree is 542 steps long (CI = 0.467; RI = 0.750; Fig 7B and S2 Fig).

Compared to the first analysis, this approach substantially increased resolution of South American gavialoid relationships. The monophyly of gryposuchines, exclusive of the Caribbean taxon *Aktiogavialis*, is recovered and *Gavialis* is identified as the sister clade of gryposuchines. Character support for the gryposuchines includes having four premaxillary alveoli (character 87–1), lack of exposure of the prootic on external braincase wall (character 164–1), a quadrate with ventromedially projected medial hemicondyle (character 181–4), and a retroarticular longitudinal crest (character 203–1). This last character is present in all South American gharials preserving this region [9,10], including an unnamed Late Oligocene gharial from Pirabas, Brazil [50]. The distinctive long posterior or posterolateral projections of the squamosal, (i.e., squamosal prongs) no longer diagnose Gryposuchinae as had been hypothesized previously (see Results in [10]), because *Argochampsa* also clearly possesses this feature [6]. Within gryposuchines, there is low support for a clade comprising *Ikanogavialis*, *Siquisiquesuchus*, and *Piscogavialis*. Other gavialoid relationships were unaffected relative to the initial analysis. Discussions below are based on this second phylogenetic analysis, unless otherwise noted.

## Discussion

### The evolutionary ecology of gavialoids: Evidence from Amazonia

*Gryposuchus pachakamue* inhabited the heart of the proto-Amazonian mega-wetlands ecosystem during the Middle Miocene, from around 16 to 13 Ma, and it represents the oldest known record of a gavialoid from this area. Remains belonging to this new taxon were consistently recovered from deposits depicting shallow lacustrine paleoenvironments. As the basalmost species of the *Gryposuchus* clade, it provides essential evidence for accurately reconstructing the ancestral anatomy and ecology of this clade and provides evidence of parallel evolution of distinctive rostral and orbital anatomy in gavialoids. Our phylogenetic analyses reconstruct the acquisition of widely separated orbits as independent evolutionary events in Asian *Gavialis* and later-diverging *Gryposuchus* species (*Gr*. *colombianus* + *Gr*. *croizati*) in South America. As a consequence, the comparatively slender interorbital bridge of *Gr*. *pachakamue* is primitive for all gavialoids (Fig 7C). A wide interorbital bridge is associated with possessing “telescoped” orbits. Traits associated with fully “telescoped” orbits, as is observed in *Gavialis* and advanced *Gryposuchus* species (but absent in *Gr*. *pachakamue*) include: postorbital bar flush with lateral jugal surface (character 135–1), upturned dorsal and posterior orbital margins (character 137–2), and ventral orbital margin with a prominent notch (character 138–1). Parsimony analyses also suggest parallel development for two of these character states, indicating that the “telescoped” orbit condition is homoplastic in gavialoids and occurred independently in advanced South American *Gryposuchus* and Asian *Gavialis* species.

**Fig 8:**
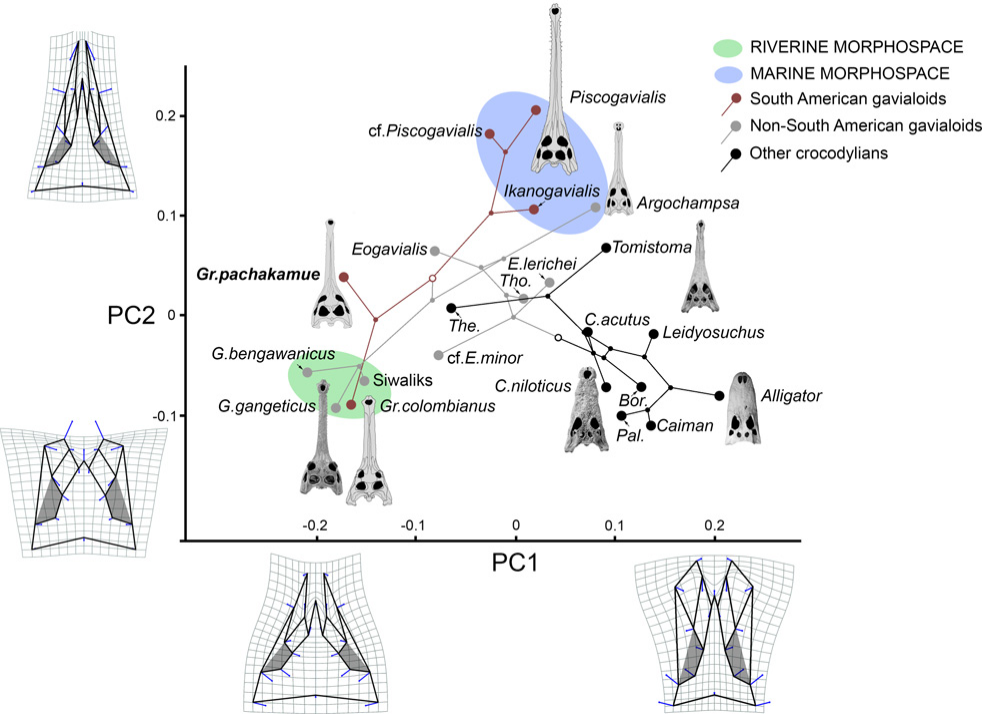
Phylogenetic relationships of Crocodylia mapped onto the circumorbital morphospace defined by PC1 and PC2. Deformation grids depict extreme values along each axis and blue vectors indicate the position of the mean relative to the landmark variation. PC1 correlates mainly with the width of the pre- and post-orbital regions, and orbit length. Species on the positive extreme of PC1 present slender skull tables and interorbital bridge, long orbits and prefrontals, and laterally oriented anterior processes of the jugals, whereas those on the negative extreme bear broad skull tables, wide posterior portion of the interorbital bridge and orbits, short orbits, short prefrontals, and medially oriented anterior processes of the jugals. PC2 correlates with the relative length of the pre-orbital bones, involving mostly the frontal and lacrimals and the width of the prefrontals. Species with higher PC2 scores have comparatively longer and more slender frontals and a narrow interorbital bridge. Taxa with lower scores present short frontal and lacrimal bones and short and wide anterior portion of the interorbital bridge. The phylogenetic morphospace of the orbital and circumorbital region in Miocene South American gavialoids covers most of the variation of the whole clade. Taxon abbreviations: *Bor*., *Borealosuchus*; *Pal*., *Paleosuchus*; *The*., *Thecachampsa*; *Tho*., *Thoracosaurus* (S1 Appendix).

By mapping the phylogenetic tree onto the first two axes of the PCA axes, we examined ecological associations of circumorbital bone arrangements throughout gavialoid evolution (Fig 8). Higher PC1 and PC2 scores define a morphospace of gavialoids found in coastal marine deposits, such as *Piscogavialis* and *Argochampsa*, whereas lower scores along these PC axes correspond to the morphospace including taxa with fully “telescoped” orbits. Lacustrine *Gryposuchus pachakamue* and *Eogavialis africanus*, the latter lacking definitive data on its paleoenvironmental provenance, exemplify the intermediate morphospace. The fully “telescoped” orbit morphospace is represented by *Gavialis* and *Gryposuchus colombianus*; since they are distantly related, this morphospace depicts the parallel evolution of this distinctive cranial anatomy, apparently associated with convergent specialization in a freshwater habitat and similar visually enhanced feeding strategies. Distinct features of this morphotype include: upturned anterior, dorsal, and posterior orbital margins; ventral orbital margin with a prominent notch; postorbital pillar laterally displaced; and orbits widely separated. Gavialoids display different degrees of the “telescoped” condition.

The feeding strategy of the extant gharial *Gavialis gangeticus* involve active use of the telescoped eyes and integumentary sense organs in capturing fishes in streams [51], as its habitat is confined to freshwater settings of the Indian subcontinent, notably restricted to riverine environments [52]. Fossil gharials with well-developed “telescoped” orbits are usually found in depositional settings documenting fluvial-dominated paleoenvironments. This similar habitat association in the extant and extinct convergent taxa offers further support for the adaptive value of possessing “telescoped” orbits and suggests that this morphotype in fossil gavialoids typically is correlated with taxa living in riverine ecosystems. Plio-Pleistocene fossil *Gavialis* species inhabited, and might have dispersed geographically via, fluvial systems then occurring between Indo-Pakistan and Southeast Asia [53]. In South America, late Middle Miocene *Gryposuchus colombianus* is restricted to fluvial-influenced settings of the Pebas Mega-Wetland System at La Venta (Colombia) [8,54] and Fitzcarrald (Peru) [11,55,56], close to the rapidly rising Andes, whereas this species is absent from coeval deposits representing brackish, dysoxic lakes, swamps and deltas of the same biome at Iquitos (Peru) where *Gryposuchus pachakamue* consistently occurs. This latter species not only is the sole gavialoid in the Iquitos fauna, but the sole longirostrine crocodylian within a highly diversified community dominated by small caimans with crushing dentitions reflecting a specialized malacophagous diet [11]. In younger beds at Iquitos (Molluscan Zone MZ9 or younger), we only recovered a “telescoped” orbit gavialoid from one locality (IQ125) in the “Uppermost Pebas Formation” [33], at a time corresponding to development of new regional fluvial-dominated conditions, attributable to a peak in Andean uplift and the onset of the Amazon River System [11].

**Fig 9:**
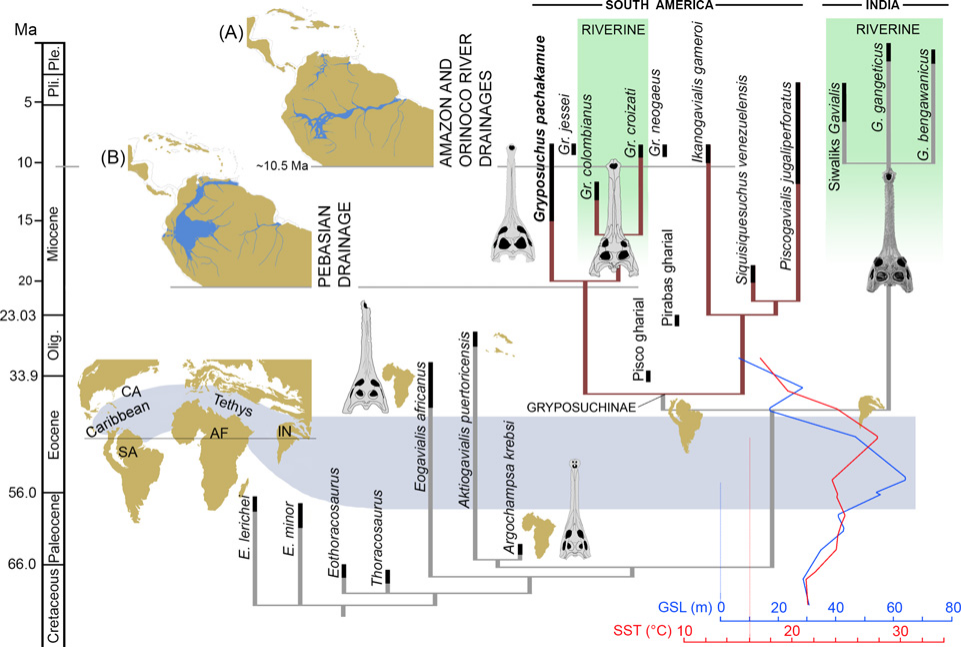
Time calibrated phylogenetic tree of the Gavialoidea and relevant paleogeographic distributions associated with the evolution and diversification of gavialoids in marine and freshwater settings. During the Late Paleocene-Early Eocene interval, peaks of sea surface temperature (SST) and global sea surface level (GSL) occurred together with tropical marine connections through the Tethys Ocean and Caribbean Sea [59,60]. During the Neogene, distinct biomes dominated tropical South America: (A) Acre Phase, after the onset of the eastern-draining Amazon and northward-draining Orinoco river systems; and (B) Pebas Mega-Wetland System, with its drainage northward to the Caribbean Sea. Abbreviations: Olig., Oligocene; Ple., Pleistocene; Pli., Pliocene. Global and South American schematic paleogeography adapted from Blakey [60] and Hoorn et al. [61], respectively.

Within a wider environmental context, the demise of the Pebas Mega-Wetland System and subsequent establishment of the fluvio-tidal Acre Phase in the Amazonian Basin at around 10.5 Ma seems to have promoted diversification, size increase, and specialization in gharials (Fig 9) compared with the presence of only one species per Middle Miocene-aged crocodylian fauna (i.e., Iquitos, La Venta, and Fitzcarrald). In contrast, Late Miocene assemblages of Acre (Brazil) contain four gavialoid taxa [57], including the conspicuous record of forms with “telescoped” orbits (RS-G, pers. obs). Although the same number of taxa is observed in the Late Miocene Urumaco Formation of Venezuela, matches between the dominant environment and specific morphotypes is far more complicated to assess there. The Urumaco Formation preserves several aquatic environments that were potential habitats for crocodylians within the Paleo-Orinoco Basin, such as delta plains, swamps, marginal marine embayments, and rivers, all with a marine influence from the Caribbean [35]. Consistent with this patchwork of environments, the Urumaco gavialoid species exhibit a highly disparate array of cranial circumorbital configurations [9,34,48]. In Urumaco a telescoped orbit specialization was fully attained by the giant *Gryposuchus croizati*, most likely a dweller in fluvial settings. Based on its paleoenvironmental preference in the Iquitos fauna, *Gryposuchus* cf. *pachakamue* from Urumaco instead might have occupied relicts of the brackish lacustrine ecosystems once widely distributed in proto-Amazonia [32]. Therefore, the record of this gavialoid at Urumaco adds evidence for the persistence of Pebasian aquatic conditions during the Late Miocene in northernmost South America, where the lower course of the Pebasian proto-Amazonian System drainage was formerly situated [11,58].

### Caribbean and proto-Amazonian biogeographic relationships and the origin of Neotropical gharials

Inasmuch as gharials were inhabitants in the Caribbean region at least since the Late Oligocene [39], the Caribbean Portal at the mouth of the proto-Amazonian aquatic system might have played an important role for the invasion of gavialoids into the inland areas. *Siquisiquesuchus* was an early Miocene Caribbean inhabitant of present-day Venezuelan coasts [10]. Other marine and fresh water units in the region document numerous remains of gharials throughout the Miocene [9,34,62,63], showing persistent faunas living close to the Caribbean Portal. Either by means of marine transgressions or via riverine drainage systems, a continuous aquatic corridor united the Caribbean Sea with western proto-Amazonia for most of the Paleogene and early Neogene [19,32,61]. The heart of proto-Amazonia during the extensive and persistent Pebas Mega-Wetland System was continuously connected with the Caribbean Portal by a northward-flowing trunk drainage [32,61]. Although not coeval, evidence of the prevailing aquatic connections that linked both areas through the Llanos (Colombia) until the Late-Middle Miocene boundary is founded on the record of *Gryposuchus pachakamue* from both Urumaco and Iquitos, as well as other biotic indicators [11,64–68]. Specific environmental conditions fostering high diversity of Late Miocene Urumaco gavialoid diversity remain elusive.

Our phylogenetic analyses propose sister-group relationships of Caribbean *Aktiogavialis* and African *Argochampsa* (Fig 7 and Fig 9). In the context of our time-calibrated phylogenetic tree, this association would suggest an African origin for the Caribbean gavialoid, probably by western transatlantic dispersal, as suggested by early authors [42,69] and more recently by Vélez-Juarbe et al. [39]. *Argochampsa*, *Aktiogavialis*, and the oldest records of South American gharials all were found in deposits from coastal marine settings [10,39,47,50,70]. Whether fossil gharials were strictly marine or not, distinct lines of evidence indicate that gavialoids flourished during high sea surface levels and temperatures (SST) of the Paleocene and Eocene epochs [59]. During this time interval (~60–45 Ma), paleogeographic reconstructions depict a tropical marine connection from India and Africa to the Caribbean and northern South America through the Tethys Ocean and Caribbean Sea [60]. Although the fossil record is far from complete in these areas, our time-calibrated phylogenetic tree and the occurrence of advanced gavialoids along both the Tethys and Caribbean coasts and islands, suggest that this marine realm could have served as a preferred habitat and dispersal system for gavialoids during the Paleogene (Fig 9). The presence of gharials in South America most likely resulted from a single transoceanic colonization event and subsequent diversification in South America, distinct from the one that gave rise to the Caribbean taxon *Aktiogavialis*.

## Conclusions

Gavialoid history exhibits independent acquisitions of the “telescoped” orbits condition. Analyses of the new Pebasian species *Gryposuchus pachakamue* and other South American fossil gavialoids document high plasticity in orbital anatomy, which appears to have been strongly correlated with a visually enhanced feeding strategy and environmental circumstances. Morphospaces occupied by fluvial and coastal marine specialists are identified by quantitative analysis of orbital and circumorbital shape variation. In light of the phylogenetic history, a fluvial habitus in South American gharials is derived from ancestral lacustrine-deltaic forms with incipient development of protruding eyes or telescoped orbits. The circumorbital region of coastal marine gavialoids is closer in morphology to that of brevirostrine crocodylians. Identifying morphological steps of parallel evolution and ancestral ecological habitus in gavialoids provides models for reconstructing puzzling phylogenetic histories and adaptive radiations within extinct crocodylomorphs clades with elongated rostrums, such as thalattosuchians, dyrosaurids, and pholidosaurids [71]. Proto-Amazonian connections with the Caribbean Sea to the north, and the subsequent onset of the transcontinental Amazon River System draining eastward, provided multiple habitats and conditions for gavialoid colonizations of new areas and extensive morphological diversification in South America throughout the mid-late Cenozoic.

## Supporting Information

S1 Appendix Phylogenetic and morphometric data. (DOCX)

S1 Fig Landmarks of the circumorbital region of *Gavialis* and its schematic representation. Landmarks are labeled from 1 to 20. Gray area symbolizes orbits defined by landmarks 5-7-9-11 and 6-8-10-12 on the right and left sides, respectively.
(TIF)

S2 Fig Strict consensus phylogenetic tree. Second approach after removing character state 137–2 and character 138. Numbers at nodes indicate Bremer support values.
(TIF)

## Abbreviations

AMNH: American Museum of Natural History, New York, USA
AMU CURS: City Hall Urumaco Municipality, Urumaco, Venezuela
IGM: INGEOMINAS, Bogotá, Colombia
MNHN: Muséum national d’Histoire naturelle, Paris, France
OCP: Office Chérifien des Phosphates, Khouribga, Morocco
SMNK: Staatliches Museum für Naturkunde Karlsruhe, Karlsrhue, Germany

## References

[1] Harshman JC, Hoffman MA, Densmore LD, Maxson LR. True and false gharials: a nuclear gene phylogeny of Crocodylia. Syst Biol. 1992; 52: 386–402. 10.1080/10635150390197028 12775527

[2] Hass CA, Hoffman MA, Densmore LD, Maxson LR. Crocodilian evolution: insights from immunological data. Molecular Phylogenet Evol. 1992; 1: 386–402. 10.1016/1055-7903(92)90015-9 1342935

[3] Gatesy JG, Amato G, Norell M, DeSalle R, Hayashi C. Total evidence support for extreme atavism in gavialine crocodylians. Syst Biol. 2003; 52: 403–422. 10.1080/10635150390197037 12775528

[4] Oaks JR. A time-calibrated species tree of Crocodylia reveals a recent radiation of the true crocodiles. Evolution 2011; 65: 3285–3297. 10.1111//j.1558-5646.2011.01373.x 22023592

[5] Brochu CA. A new Late Cretaceous gavialoid crocodylian from eastern North America and the phylogenetic relationships of thoracosaurs. J Vertebr Paleontol. 2004; 24: 610–633. 10.1671/0272-4634(2004)024[0610:ANLCGC]2.0.CO;2

[6] Jouve S, Bardet N, Jalil N-E, Suberbiola XP, Bouya B, Amaghzaz M. The oldest African crocodylian: phylogeny, paleobiogeography, and differential survivorship of marine reptiles through the Cretaceous-Tertiary boundary. J Vertebr Paleontol. 2008; 28: 409–421. 10.1671/0272-4634(2008)28[409:TOACPP]2.0.CO;2

[7] Clark JM. Patterns of evolution in Mesozoic crocodyliformes In: Fraser NC, Sues H-D, editors. In the Shadow of the Dinosaurs: Early Mesozoic Tetrapods. New York: Cambridge University Press; 1994 pp. 84–97.

[8] Langston W Jr, Gasparini Z. Crocodilians, *Gryposuchus*, and the South American gavials In: Kay RF, Madden RH, Cifelli RL, Flynn JJ, editors. Vertebrate Paleontology in the Neotropics–The Miocene fauna of La Venta, Colombia. Washington DC: Smithsonian Institution; 1997 pp. 113–154.

[9] Riff D, Aguilera OA. The world’s largest gharials *Gryposuchus*: description of *G*. *croizati* n. sp. (Crocodylia, Gavialidae) from the Upper Miocene Urumaco Formation, Venezuela. Paläont Z. 2008; 82: 178–195. 10.1007/BF02988408

[10] Brochu CA, Rincón AD. A gavialoid from the lower Miocene of Venezuela. Spec pap Palaeont. 2004; 71: 61–79.

[11] Salas-Gismondi R, Flynn JJ, Baby P, Tejada-Lara JV, Wesselingh FP, Antoine P-O. A Miocene hyperdiverse crocodylian community reveals peculiar trophic dynamics in proto-Amazonian mega-wetlands. Proc R Soc B 2015; 282: 20142490 10.1098/rspb.2014.2490 25716785 PMC4375856

[12] Wesselingh FP, Salo JA. A Miocene perspective on the evolution of the Amazonian biota. Scr Geol. 2006; 133: 439–458.

[13] Brochu CA. Phylogenetics, taxonomy, and historical biogeography of Alligatoroidea. Soc Vertebr Paleontol Mem. 1999; 6: 9–100. 10.1080/02724634.1999.10011201

[14] Brochu CA. Phylogenetic relationships of *Necrosuchus ionensis* Simpson, 1937 and the early history of caimanines. Zool J Linn Soc. 2011; 163: 228–256. 10.1111/j.1096-3642.2011.00716.x

[15] Goloboff P, Farris J, Nixon K. T.N.T, a free program for phylogenetic analysis. Cladistics 2008; 24: 744–786. 10.1111/j.1096-0031.2008.00217.x

[16] Mook CC. Individual and age variations in the skull of recent crocodilia. Bull Am Mus Nat Hist. 1921; 44: 51–66.

[17] Claude J. Morphometrics with R. New York: Springer; 2008.

[18] Adams DC, Otarola-Castillo E. Geomorph: an R package for the collection and analysis of geometric morphometric shape data. Methods Ecol Evol. 2014; 4: 393–399. 10.1111/j.1096-0031.2008.00217.x

[19] R Development Core Team. R: A Language and Environment for Statistical Computing. Vienna, Austria: the R Foundation for Statistical Computing; 2011 ISBN: 3-900051-07-0. Accessed: http://www.R-project.org/

[20] Rohlf FJ. Shape statistics: Procrustes superimpositions and tangent spaces. J Classif. 1999; 16: 197–223.

[21] Klingenberg CP. MorphoJ: an integrated software package for geometric morphometrics. Mol Ecol Resour. 2011; 11: 353–357. 10.1111/j.1755-0998.2010.02924.x 21429143

[22] Hovikoski J. Sedimentology, ichnology and sequence stratigraphy of four outcrops of the Early-Late Miocene Pebas Formation, Western Amazonian foreland basin, Peru. M.Sc. Thesis, University of Turku, Finland; 2001.

[23] Gingras MK, Räsänen ME, Pemberton SG, Romero LP. Ichnology and sedimentology reveal depositional characteristics of bay-margin parasequences in the Miocene Amazonian foreland basin. J Sediment Res. 2002; 72: 871–883.

[24] Roddaz M, Baby P, Brusset S, Hermoza W, Darrozes JM. Forebulge dynamics and environmental control in Western Amazonia: The case study of the Arch of Iquitos (Peru). Tectonophysics 2005; 399: 87–108. 10.1016/j.tecto.2004.12.017

[25] Hoorn C, Wesselingh FP, Hovikoski J, Guerrero J. The development of the Amazonian mega-wetland (Miocene; Brazil, Colombia, Peru, Bolivia) In: Hoorn C, Wesselingh FP, editors. Amazonia, landscape and species evolution. Oxford: Wiley-Blackwell; 2010 pp. 123–142.

[26] Hoorn MC. Marine incursions and the influences of Andean tectonics on the Miocene depositional history of northwestern Amazonia: results of a palynostratigraphic study. Palaeogeogr Palaeoclimat Palaeoecol. 1993; 105: 267–309.

[27] Muñoz-Torres FA, Whatley RC, van Harten D. Miocene ostracod (Crustacea) biostratigraphy of the upper Amazon Basin and evolution of the genus *Cyprideis*. J S Am Earth Sci. 2006; 21: 75–86. 10.1016/j.james.2005.08.005

[28] Wesselingh FP, Hoorn MC, Guerrero J, Räsänen ME, Romero Pittmann L, Salo JA. The stratigraphy and regional structure of Miocene deposits in western Amazonia (Peru, Colombia and Brazil), with implications for late Neogene landscape evolution. Scr Geol. 2006; 133: 291–322.

[29] Nuttall CP. A review of the Tertiary non-marine molluscan faunas of the Pebasian and other inland basins of north-western South America. Bull brit Mus nat Hist Geol. 1990; 45: 165–371.

[30] Hoorn C. An environmental reconstruction of the palaeo-Amazon River system (Middle-Late Miocene, NW Amazonia). Palaeogeogr Palaeoclimatol Palaeoecol. 1994; 112: 187–238.

[31] Wesselingh FP, Räsänen ME, Irion G, Vonhof HB, Kaandorp R, Renema W, Romero Pittman L, Gingras M. Lake Pebas: a palaeoecological reconstruction of a Miocene, long-lived lake complex in western Amazonia. Cainoz Res. 2002; 1: 35–81.

[32] Boonstra M, Ramos MIF, Lammertsma EI, Antoine P-O, Hoorn C. Marine connections of Amazonia: Evidence from foraminifera and dinoflagellate cysts (early to middle Miocene, Colombia/Peru). Palaeogeogr Palaeoclimatol Palaeoecol. 2015; 417: 176–194. 10.1016/j.palaeo.2014.10.032

[33] Rebata LA, Räsänen ME, Gingras MK, Vieira V Jr, Berberi M, Irion G. Sedimentology and ichnology of tide-influenced Late Miocene successions in western Amazonia: the gradational transition between the Pebas and Nauta formations. J S Am Earth Sci. 2006; 21: 116–129. 10.10116/jsames.2005.07.011

[34] Sánchez-Villagra MR, Aguilera OA. Neogene vertebrates from Urumaco, Falcón State, Venezuela: diversity and significance. J Syst Palaeontol. 2006; 4: 213–220. 10.1017/s1477201906001829

[35] Quiroz LI, Jaramillo CA. Stratigraphy and sedimentary environment of Miocene shallow to marginal marine deposits in the Urumaco trough, Falcon Basin, Western Venezuela In: Sánchez-Villagra M, Aguilera OA, Carlini AA, editors. Urumaco & Venezuelan Paleontology. Bloomington: Indiana University Press; 2010 pp. 153–172. 10.1007/s13358-015-0071-4

[36] Hay OP. Second bibliography and catalogue of the fossil vertebrata of North America. Carnegie I Wash, 1930; II: 1–415.

[37] Huxley TH. On *Stagonolepis robertsoni*, and on the evolution of Crocodilia. Q J Geol Soc. 1875; 31: 423–438.

[38] Gmelin J. Linnei Systema Naturae. Leipzig; 1789.

[39] Velez-Juarbe J, Brochu CA, Santos H. A gharial from the Oligocene of Puerto Rico: transoceanic dispersal in the history of a non-marine reptile. Proc R Soc B 2007; 274: 1245–1254. 10.1098/rspb.2006.0455 17341454 PMC2176176

[40] Gürich GJE. *Gryposuchus jessei*: ein neues schmalschnauziges Krokodil aus den jüngeren ablagerungen des oberen Amazonas-Gebietes. Jahrb Hamburg Wise Anat. 1912; XXIX: 59–71.

[41] Vargas-Llosa M. El Hablador, Lima, Perú: Grupo Planeta; 1987.

[42] Buffetaut E. Systématique, origine et evolution des Gavialidae Sud-Américaines. Geobios 1982; 6: 127–140.

[43] Norell MA. The higher level relationship of the extant Crocodylia. J Herpetol. 1989; 23: 325–335.

[44] Gasparini Z. Nuevos restos de *Rhamphostomopsis neogaeus* (Burm.) Rusconi 1933, (Reptilia, Crocodilia) del “Mesospotamiense” (Plioceno medio-superior) de Argentina. Ameghiniana 1968; 8: 299–311.

[45] Kraus R. The cranium of *Piscogavialis jugaliperforatus* n.gen., n.sp. (Gavialidae, Crocodylia) from the Miocene of Peru. Paläontol Zeitschr. 1998; 72: 389–406.

[46] Kälin JS. Beitrage zur vergleichenden Osteologie des Crocodilidenschädels. Zool Jahrb. 1933; 57: 535–714.

[47] Jouve S, Iarochène M, Bouya B, Amaghzaz M. New material of *Argochampsa krebsi* (Eusuchia: Gavialoidea) and phylogenetic implications. Geobios 2006; 39: 817–832.

[48] Sill WD. Nota preliminar sobre un nuevo gavial del Plioceno de Venezuela y una discusión de los gaviales sudamericanos. Ameghiniana 1970; 7: 151–159.

[49] Brochu CA. Osteology and phylogenetic significance of *Eosuchus minor* (Marsh, 1970) new combination, a longirostrine crocodylian from the Paleocene of North America. J Paleont. 2006; 80: 162–186. 10.1666/0022-360(2006)080[0162:OAPSOE]2.0.CO;2

[50] Moraes-Santos H, Bocquentin-Villanueva J, Toledo PM. New remains of a gavialoid crocodilian from the late Oligocene-early Miocene of the Pirabas Formation, Brazil. Zool J Linn Soc. 2011; 163: S132–S139. 10.1111/j.1096-3642.2011.00710.x

[51] Thorbjarnarson JB. Notes on the feeding behavior of the gharial (*Gavialis gangeticus*) under semi-natural conditions. J Herpetol. 1990; 24: 99–100.

[52] Whitaker R, Basu D. The gharial (*Gavialis gangeticus*): a review. J Bombay Nat Hist Soc. 1983; 79: 531–548.

[53] Martin JE, Buffetaut E, Naksri W, Lauprasert K, Claude J. *Gavialis* from the Pleistocene of Thailand and its relevance for drainage connections from India to Java. PLoS ONE 2012; 7(9): e44541 10.1371/journal.pone.oo44541 23028557 PMC3445548

[54] Langston W Jr. Fossil crocodilians from Colombia and the Cenozoic history of the Crocodilia in South America. U Calif Publ Geol Sci. 1965; 52: 1–157.

[55] Salas-Gismondi R, Antoine P-O, Baby P, Brusset S, Benammi M, Espurt N et al Middle Miocene crocodiles from the Fitzcarrald Arch, Amazonian Peru. Cuadernos del Museo Geominero 2007; 8: 355–360.

[56] Tejada-Lara JV, Salas-Gismondi R, Pujos F, Baby P, Benammi M, Brusset S et al Life in proto-Amazonia: Middle Miocene mammals from the Fitzcarrald Arch (Peruvian Amazonia). Palaeontology 2015; 1–38. 10.1111/pala.12147

[57] Cozzuol MA. The Acre vertebrate fauna: age, diversity, and geography. J S Am Earth Sci. 2007; 21: 185–203. 10.1016/jsames.2006.03.005

[58] Aguilera O, Lundberg J, Birindelli J, Perez MS, Jaramillo C, Sánchez-Villagra MR. Palaeontological evidence for the last temporal occurrence of the ancient western Amazonian River outflow into the Caribbean. PLoS ONE 2013; 8(9): e76202 10.1371/journal.pone.0076202 24098778 PMC3786985

[59] Martin JE, Amiot R, Lécuyer C, Benton MJ. Sea surface temperature contributes to marine crocodyliform evolution. Nat Commun. 2014; 5: 4658 10.1038/ncomms5658 25130564

[60] Blakey RC. Gondwana paleogeography from assembly to breakup—A 500 m.y. odyssey. In: Fielding CR, Frank TD, Isbell JL, editors. Resolving the Late Paleozoic Ice Age in Time and Space. Boulder: Geological Society of America Special Paper 441; 2008. pp. 1–28.

[61] Hoorn C, Wesselingh FP, Steege H, Bermudez MA, Mora A, Sevink J et al Amazonia through time: Andean uplift, climate change, landscape evolution, and biodiversity. Science 2010; 330: 927–931. 10.1126/science.1194585 21071659

[62] Scheyer TM, Aguilera OA, Delfino M, Fortier DC, Carlini AA, Sánchez R, et al Crocodylian diversity peak and extinction in the late Cenozoic of the northern Neotropics. *Nat*. *Commun*. 2013; 4:1907 10.1038/ncomms2940 23695701

[63] Moreno F, Hendy JW, Quiroz L, Hoyos N, Jones DS, Zapata V et al Revised stratigraphy of Neogene strata in the Cocinetas Basin, La Guajira, Colombia. Swiss J Palaeontol. 2015; 134: 5–43. 10.1007/s13358-015-0071-4

[64] Nuttall CP. A review of the Tertiary non-marine molluscan faunas of the Pebasian and other inland basins of north-western South America. Bull br Mus nat Hist. 1990; 45: 165–371.

[65] Hoorn C. Marine incursions and the influence of Andean tectonics on the Miocene depositional history of northwestern Amazonia: results of a palynostratigraphic study. Palaeogeogr Palaeoclimatol Palaeoecol. 1993; 109: 267–309. 10.1016/0031-0182(93)90087-Y

[66] Lundberg JG, Marshall LG, Guerrero J, Horton B, Malabarba MCSL, Wesselingh F. The stage for Neotropical fish diversification: a history of tropical South American rivers In: Malabarba LR, Reiss RE, Vari RP, Lucena ZM, Lucena CAS, editors. Phylogeny and Classification of Neotropical Fishes. Porto Alegre: EDIPUCRS; 1998 pp. 13–48.

[67] Lovejoy NR, Bermingham E, Martin AP. Marine incursion into South America. Nature 1998; 396: 421–422. 10.1038/24757

[68] Wesselingh FP, Macsotay O. *Pachydon hettneri* (Anderson, 1928) as indicator for Caribbean-Amazonian lowland connections during the Early-Middle Miocene. J S Am Earth Sci. 2006; 21: 49–53.

[69] Bocquentin-Villanueva J, Buffetaut E. *Hesperogavialis cruxenti* n. gen. n. sp., nouveau gavialidae (Crocodylia, Eusuchia) du Miocéne supérieur (Huayquerian) d’Urumaco (Venezuela). Geobios 1981; 14: 415–419.

[70] Hua S, Jouve S. A primitive marine gavialoid from the Paleocene of Morocco. J Vertebr Paleontol. 2004; 24: 341–350. 10.1671/1104

[71] Wilberg EW. What’s in an outgroup? The impact of outgroup choice on the phylogenetic position of Thalattosuchia (Crocodylomorpha) and the origin of Crocodyliformes. Syst Biol. 2015; 64: 621–637. 10.1093/sysbio/syv020 25840332

