# Supplementary material for: A New 13 Million Year Old Gavialoid Crocodylian from Proto-Amazonian Mega-Wetlands Reveals Parallel Evolutionary Trends in Skull Shape Linked to Longirostry
Source: PLoS One. 2016 Apr 20;11(4):e0152453. doi: 10.1371/journal.pone.0152453 (PMC4838223; doi:10.1371/journal.pone.0152453)
Supplement: S1 Appendix — (DOCX) [file pone.0152453.s001.docx]

**(1) Additional phylogenetic data and results**

**Character list**

Skull and mandibles. This character list was developed primarily by Brochu [1,2] and Jouve [3]. A small number of characters are derived from previous contributions [4-18] Modifications/additions and new characters are indicated in bold font. Three characters are entirely new (i.e., 202-204). This list includes only osteological characters from the skull and jaws. For characters 1-46 see Brochu [1].

47. Alveoli for dentary teeth 3 and 4 nearly same size and confluent (0), or fourth alveolus larger than third, and alveoli are separated (1).

48. Anterior dentary teeth strongly procumbent (0) or project anterodorsally (1).

49. Dentary symphysis extends to fourth or fifth alveolus (0); or sixth to eighth alveolus (1); **or eighth to twelfth alveolus (2); or beyond twelfth (3). Modified from Brochu [18], character 166.**

50. Dentary gently curved (0), or deeply curved (1), or linear (2) between fourth and tenth alveoli.

51. Largest dentary alveolus immediately caudal to fourth is 13 or 14 (0); 11 or 12 (1); no differentiation (2); or behind 14 (3).

52. Splenial with anterior perforation for mandibular ramus of cranial nerve V (0) or lacks anterior perforation for mandibular ramus of cranial nerve V (1).

53. Mandibular ramus of cranial nerve V exits splenial anteriorly only (0) or splenial has singular perforation for mandibular ramus of cranial nerve V posteriorly (1) or splenial has double perforation for mandibular ramus of cranial nerve V posteriorly (2).

54. Splenial participates in mandibular symphysis; splenial symphysis adjacent to no more than five dentary alveoli (0), or splenial excluded from mandibular symphysis; anterior tip of splenial passes ventral to Meckelian groove (1), or splenial excluded from mandibular symphysis; anterior tip of splenial passes dorsal to Meckelian groove (2), or deep splenial symphysis, longer than five dentary alveoli; splenial forms wide V within symphysis (3), or deep splenial symphysis, longer than five dentary alveoli; splenial constricted within symphysis and forms narrow V (4).

55. Coronoid bounds posterior half of foramen intermandibularis medius (0), or completely surrounds foramen intermandibularis medius at maturity (1), or obliterates foramen intermandibularis medius (2) at maturity.

56. Superior edge of coronoid slopes strongly anteriorly (0) or almost horizontal (1).

57. Inferior process of coronoid laps strongly over inner surface of Meckelian fossa (0), or remains largely on medial surface of mandible (1).

58. Coronoid imperforate (0), or with perforation posterior to foramen intermandibularis medius (1).

59. Process of splenial separates angular and coronoid (0) or no splenial process between angular and coronoid (1).

60. Angular–surangular suture contacts external mandibular fenestra at posterior angle at maturity (0) or passes broadly along ventral margin of external mandibular fenestra late in ontogeny (1).

61. Anterior processes of surangular unequal, little or no ventral process (0) or subequal to equal, well development ventral process (1).

62. Surangular with spur bordering the dentary tooth row lingually for at least one alveolus length (0), or lacking such spur (1).

63. External mandibular fenestra absent (0), or present (1), or present and very large; most of foramen intermandibularis caudalis visible in lateral view (2).

64. Surangular–dentary suture intersects external mandibular fenestra anterior to posterodorsal corner (0), or at posterodorsal corner (1).

65. Angular extends dorsally toward or beyond anterior end of foramen intermandibularis caudalis; anterior tip acute (0) or, does not extend dorsally beyond anterior end of foramen intermandibularis caudalis; anterior tip very blunt.

66. Surangular–angular suture lingually meets articular at ventral tip (0), or dorsal to tip.

67. Surangular continues to dorsal tip of lateral wall of glenoid fossa (0), or truncated and not continuing dorsally (1).

68. Articular–surangular suture simple (0), or articular bears anterior lamina dorsal to lingual foramen (1), or articular bears anterior lamina ventral to lingual foramen (2), or bears laminae above and below foramen (3).

69. Lingual foramen for articular artery and alveolar nerve perforates surangular entirely (0), or perforates surangular-articular suture (1).

70. Foramen aerum at extreme lingual margin of retroarticular process (0), or set in from margin of retroarticular process (1).

71. Retroarticular process projects posteriorly (0), projects posterodorsally, not higher than the posterior edge of the articular fossa (1), or projects posterodorsally higher than the posterior edge of the articular fossa (2).

72. Surangular extends to posterior end of retroarticular process (0), or pinched off anterior to tip of retroarticular process (1).

73. Surangular–articular suture orientated anteroposteriorly (0), or bowed strongly laterally within glenoid fossa (1).

74. Sulcus between articular and surangular (0), or articular flush against surangular within the adductor fossa (1).

For characters 75-78 see Brochu [1].

79. Teeth and alveoli of maxilla and/or dentary circular in cross-section (0), or posterior teeth laterally compressed (1), or all teeth compressed (2).

80. Maxillary and dentary teeth with smooth carinae (0), or serrated (1), or with neither carinae nor serrations (2).

81. Naris projects anterodorsally (0), or dorsally (1).

82. External naris bisected by nasals (0), or nasals contact external naris, but do not bisect it (1), or nasals excluded, at least externally, from naris; nasals and premaxillae still in large contact (2), or nasals excluded from naris and nasals and premaxillae in weak contact (3), or nasals and premaxillae not in contact (4).

83. Naris circular or keyhole-shaped (0), or wider than long (1), or anteroposteriorly long and prominently teardrop-shaped (2).

84. External naris of reproductively mature males remains similar to that of females (0), or develops bony excrescence (ghara) (1).

85. External naris opens flush with dorsal surface of premaxillae (0), or circumscribed by a crest (1).

86. Premaxillary surface lateral to naris smooth (0), or with deep notch lateral to naris (1).

87. Premaxilla has five teeth (0) or four teeth (1) early in posthatching ontogeny.

88. Incisive foramen small, less than half the greatest width of premaxillae (0), or extremely reduced and thin (1), or large, more than half the greatest width of premaxillae (2), or large, and intersects premaxillary–maxillary suture (3).

89. Incisive foramen completely situated far from premaxillary tooth row, at the level of the second or third alveolus (0), or abuts premaxillary tooth row (1), or projects between first premaxillary teeth (2).

90. Dorsal premaxillary processes short, not extending beyond third maxillary alveolus (0), or long, extending beyond third maxillary alveolus (1).

91. Dentary tooth 4 occludes in notch between premaxilla and maxilla early in ontogeny (0), or occludes in a pit between premaxilla and maxilla; no notch early in ontogeny (1).

92. All dentary teeth occlude lingual to maxillary teeth (0), or occlusion pit between seventh and eighth maxillary teeth; all other dentary teeth occlude lingually (1), or dentary teeth occlude in line with maxillary tooth row (2).

93. Largest maxillary alveolus is no. 3 (0), or no. 5 (1), or no. 4 (2), or nos. 4 and 5 are same size (3), or no. 6 (4), or maxillary teeth homodont (5), or maxillary alveoli gradually increase in diameter posteriorly toward penultimate alveolus (6).

94. Maxillary toothrow posterior to first six maxillary alveoli curved medially or linear (0), or curves laterally broadly (1).

95. Dorsal surface of rostrum curves smoothly (0), or bears medial dorsal boss (1).

96. Canthi rostralii absent or very modest (0), or very prominent at maturity (1).

97. Preorbital ridges absent or very modest (0), or very prominent at maturity (1).

98. Antorbital fenestra present (0), or absent (1).

99. Vomer entirely obscured by premaxilla and maxilla (0), or exposed on palate at premaxillary–maxillary suture (1).

100. Vomer entirely obscured by maxillae and palatines (0), or exposed on palate between palatines (1).

101. Surface of maxilla within narial canal imperforate (0), or with a linear array of pits (1).

102. Medial jugal foramen small (0), or very large (1).

103. Maxillary foramen for palatine ramus of cranial nerve V small or not present (0), or very large (1).

104. Ectopterygoid abuts maxillary tooth row (0), or maxilla broadly separates ectopterygoid from maxillary tooth row (1).

105. Maxilla terminates in palatal view anterior to lower temporal bar (0), or comprises part of the lower temporal bar (1).

106. Penultimate maxillary alveolus less than twice the diameter of the last maxillary alveolus (0), or more than twice the diameter of the last maxillary alveolus (1).

107. Prefrontal dorsal surface smooth adjacent to orbital rim (0) or bearing discrete knob-like processes (1).

108. Dorsal half of prefrontal pillar narrow (0) or expanded anteroposteriorly (1).

109. Medial process of prefrontal pillar expanded dorsoventrally (0) or anteroposteriorly (1).

110. Prefrontal pillar solid (0) or with large pneumatic recess (1).

111. Medial process of prefrontal pillar wide (0) or constricted at base (1).

112. Maxilla has linear medial margin adjacent to suborbital fenestra (0) or bears broad shelf extending into fenestra, making lateral margin concave (1).

113. Anterior face of palatine process rounded or pointed anteriorly (0) or notched anteriorly (1).

114. Anterior ectopterygoid process tapers to a point (0) or forked (1).

115. Palatine process extends (0) or does not extend (1) significantly beyond anterior end of suborbital fenestra.

116. Palatine process generally broad anteriorly (0) or in form of thin wedge (1).

117. Lateral edges of palatines smooth anteriorly (0) or with lateral process projecting from palatines into suborbital fenestrae (1).

118. Palatine–pterygoid suture nearly at (0), or far anteriorly from posterior angle (1) of suborbital fenestra.

119. Pterygoid ramus of ectopterygoid straight, posterolateral margin of suborbital fenestra linear (0) or ramus bowed, posterolateral margin of fenestra concave (1).

120. Lateral edges of palatines parallel posteriorly (0) or flare posteriorly, producing shelf (1).

121. Anterior border of the choana is comprised of the palatines (0) or choana entirely surrounded by pterygoids (1).

122. Choana projects posteroventrally (0) or anteroventrally (1) at maturity.

123. Pterygoid surface lateral and anterior to internal choana flush with choanal margin (0) or pushed inward anterolateral to choanal aperture (1).

124. Posterior rim of internal choana not deeply notched (0), or deeply notched (1).

125. Internal choana not septated (0), or with septum that remains recessed within choana (1), or with septum that projects out of choana (2).

126. Ectopterygoid–pterygoid flexure disappears during ontogeny (0), or remains throughout ontogeny (1).

127. Ectopterygoid extends (0), or does not extend (1) to posterior tip of lateral pterygoid flange at maturity.

128. No posterior process of maxilla within lacrimal or within lacrimal and prefrontal (0), or maxilla with posterior process within lacrimal (1), or maxilla with posterior process between lacrimal and prefrontal (2).

129. Prefrontals separated by the frontal and nasals, anterior process of frontal extending far anterior to the anterior margin of the orbit (0), prefrontals separated by the frontal and nasals, anterior process of frontal around the same level or posterior to the anterior margin of the orbit (1), or prefrontals meet medially, anterior process of frontal around the same level or posterior to the anterior margin of the orbit (2).

130. Lacrimal longer than prefrontal (0), or prefrontal longer than lacrimal (1), or lacrimal and prefrontal both elongate and nearly the same length (2).

131. Anterior tip of frontal forms simple acute point (0), or forms broad, complex sutural contact either with the nasals or prefrontals (1).

132. Ectopterygoid extends along medial face of postorbital bar (0), or stops abruptly ventral to postorbital bar (1).

133. Postorbital bar massive and anteroposteriorly oval in cross section (0), or slender and rounded in cross section (1).

134. Postorbital bar bears process that is prominent, dorsoventrally broad, and divisible into two spines (0), or bears process that is short and generally not prominent (1).

135. Ventral margin of postorbital bar flush with lateral jugal surface (0), or inset from lateral jugal surface (1).

136. Postorbital bar continuous with anterolateral edge of skull table (0), or inset (1).

137. Dorsal margin of orbit flush with skull surface (0), or dorsal edges of orbits upturned, or (2) **dorsal and posterior edges upturned**. **Modified from Brochu, [2], character 103.**

138. Ventral margin of the orbit gently circular (0) or with a prominent notch (1).

139. Palpebral forms from single ossification (0), or from multiple ossifications (1).

140. Quadratojugal spine prominent at maturity (0) or greatly reduced or absent at maturity (1).

141. Quadratojugal spine low, near posterior angle of infratemporal fenestra (0), or high, between posterior and superior angles of infratemporal fenestra (1).

142. Quadratojugal forms posterior angle of infratemporal fenestra (0), jugal forms posterior angle of infratemporal fenestra (1), or quadratojugal–jugal suture lies at posterior angle of infratemporal fenestra (2).

143. Postorbital neither contacts quadrate nor quadratojugal medially (0), or contacts quadratojugal, but not quadrate, medially (1), or contacts quadrate and quadratojugal at dorsal angle of infratemporal fenestra (2), or contacts quadratojugal with significant descending process (3).

144. Quadratojugal bears long anterior process along lower temporal bar (0), or bears modest process, or none at all, along lower temporal bar (1).

145. Quadratojugal extends to superior angle of infratemporal fenestra (0), or does not extend to superior angle of infratemporal fenestra; quadrate participates in fenestra (1).

146. Postorbital–squamosal suture orientated ventrally (0), or passes medially ventral to skull table (1).

147. Dorsal and ventral rims of squamosal groove for external ear valve musculature parallel (0), or squamosal groove flares anteriorly (1).

148. Squamosal–quadrate suture extends dorsally along posterior margin of external auditory meatus (0), or extends only to posteroventral corner of external auditory meatus (1).

149. Posterior margin of otic aperture smooth (0), or bowed (1).

150. Frontoparietal suture deeply within supratemporal fenestra; frontal prevents broad contact between postorbital and parietal (0), or suture makes modest entry into supratemporal fenestra at maturity; postorbital and parietal in broad contact (1), or suture on skull table entirely (2).

151. Frontoparietal suture between supratemporal fenestrae concavoconvex (0), or linear (1).

152. Supratemporal fenestra with fossa; dermal bones of skull roof do not overhang rim at maturity (0), or dermal bones of skull roof overhang rim of supratemporal fenestra near maturity (1), or supratemporal fenestra closes during ontogeny (2).

153. Shallow fossa at anteromedial corner of supratemporal fenestra (0), or no such fossa; anteromedial corner of supratemporal fenestra smooth (1).

154. Medial parietal wall of supratemporal fenestra imperforate (0), or bearing foramina (1).

155. Parietal and squamosal widely separated by quadrate on posterior wall of supratemporal fenestra (0), or parietal and squamosal approach each other on posterior wall of supratemporal fenestra without actually making contact (1), parietal and squamosal meet along posterior wall of supratemporal fenestra (2).

156. Skull table surface slopes ventrally from sagittal axis (0), or planar at maturity (1).

157. Squamosal on skull table is horizontal or nearly so (0), or upturned to form a posterolateral discrete horn (1); or producing a high transversely oriented eminence at the posterior margin (2) late in ontogeny.

158. Mature skull table with broad curvature; short posterolateral squamosal rami along paroccipital process (0), or with nearly straight sides; significant posterolateral squamosal rami along paroccipital process (1), or with nearly straight sides; posterolateral squamosal processes form long “prongs” (2).

159. Squamosal does not extend (0), or extends ventrolaterally to lateral extent of paraoccipital process (1).

160. Supraoccipital exposure on dorsal skull table small (0), or points posteriorly to the caudal margin of the parietal (1), or absent (2), or large (but parietals still in posterior border) (3), or large such that parietal is excluded from posterior edge of table (4).

161. Anterior foramen for palatine ramus of cranial nerve VII ventrolateral (0), or ventral (1) to basisphenoid rostrum.

162. Sulcus on anterior braincase wall lateral to basisphenoid rostrum (0), or braincase wall lateral to basisphenoid rostrum smooth; no sulcus (1).

163. Basisphenoid not exposed extensively (0), or exposed extensively (1), on braincase wall anterior to trigeminal foramen.

164. Extensive exposure of prootic on external braincase wall (0), or prootic largely obscured by quadrate and laterosphenoid externally (1).

165. Laterosphenoid bridge comprised entirely of laterosphenoid (0), or with ascending process of palatine (1).

166. Capitate process of laterosphenoid orientated laterally (0), or anteroposteriorly toward midline (1).

167. Parietal with recess communicating with pneumatic system (0), or solid, without recess (1).

168. Significant ventral quadrate process on lateral braincase wall (0), or quadrate–pterygoid suture linear from basisphenoid exposure to trigeminal foramen (1).

169. Lateral carotid foramen opens lateral (0), or dorsal (1) to basisphenoid at maturity.

170. External surface of basioccipital ventral to occipital condyle orientated posteroventrally (0) or posteriorly (1) at maturity.

171. Posterior pterygoid processes tall and prominent (0), or small and project posteroventrally (1), or small and project posteriorly (2).

172. Basisphenoid thin (0), or anteroposteriorly wide (1) ventral to basioccipital.

173. Basisphenoid not broadly exposed ventral to basioccipital at maturity; pterygoid short ventral to median eustachian opening (0), or basisphenoid exposed as broad sheet ventral to basioccipital at maturity; pterygoid tall ventral to median eustachian opening (1).

174. Exoccipital with very prominent boss on paroccipital process; process lateral to cranioquadrate opening short (0), or exoccipital with small or no boss on paroccipital process; process lateral to cranioquadrate opening long (1).

175. Lateral eustachian canals open dorsal (0) or lateral (1) to medial eustachian canal.

176. Exoccipitals terminate dorsal to basioccipital tubera (0), or send robust process ventrally and participate in basioccipital tubera (1), or send slender process ventrally to basioccipital tubera (2).

177. Quadrate foramen aerum on mediodorsal angle (0), or on dorsal surface of quadrate (1).

178. Quadrate foramen aerum is small (0), or comparatively large (1), or absent (2) at maturity.

179. Quadrate lacks (0), or bears (1) prominent, mediolaterally thin crest on dorsal surface of ramus.

180. Attachment scar for posterior mandibular adductor muscle on ventral surface of quadrate ramus forms modest crests (0), or prominent knob (1).

181. Quadrate with small, ventrally reflected medial hemicondyle (0), or with small medial hemicondyle; dorsal notch for foramen aereum (1), or with prominent dorsal projection between hemicondyles (2), or with expanded medial hemicondyle (3), **or more detached, ventromedially projected medial hemicondyle (4).** **From Riff & Aguilera [4], after Brochu [2], character 112.**

182. Edge of the maxillary tooth alveoli lower or at the same level than the space between toothrow (0) or edge of maxillary tooth alveoli higher than the space between toothrow (toothrow underlined) (1).

183. Ventral border of exoccipital: convex and ventrally projected, hiding the posterior opening of the cranioquadrate passage from the occipital view (0), or straight, sharpen or smoothly convex and does not hide the posterior opening of the cranioquadrate passage from the occipital view (1).

184. Occipital surface sloped, visible in dorsal view (0), or vertical or not visible in dorsal view (1) at maturity.

185. **Ventral premaxillary-maxillary suture mainly transversal to W-shaped (0), or acute, V-shaped suture, exceeds posteriorly the level of the second alveoli (1). Modified from Jouve [17], character 168.**

186. Less than 18 teeth (0), 18 to 22 teeth (1), or more than 22 teeth (2) on maxilla.

187. Lateral edge of the skull table at the level of the postorbital-squamosal suture situated laterally or at the same level as (0), or medially to (1) the quadrate condyle in dorsal view at maturity.

188. Frontal ends at the same level or posterior (0) or extends well anterior (1) to the anterior extension of the prefrontal.

189. Maxilla posterior process without tooth, short or absent (0), or long, longer to the distance between the three last teeth (1) in ventral view.

190. **Interorbital bridge narrower to equivalent (0), or broader (1) than the width of the orbit. Modified from Jouve [17], character 181.**

191. **Supratemporal fenestra longer than wide, rounded (0), or quadrangular, wider than long, large (1) at maturity. Jouve et al. [3], character 199.**

192. Presence (0), or absence (1) of a medial crest on the basioccipital.

193. Absence (0), or presence (1) of a posterior dentary process between splenial and angular on the ventral side.

194 Dorsal margin of the articular on the retroarticular process largely visible in lateral view (0), or slightly or not visible in lateral view (1).

195 Posterior margin of the orbit anterior to the posterior margin of the suborbital fenestra (0), or posterior or at the same level than the posterior margin of the suborbital fenestra (1) measured at the level of the postorbital-frontal suture in the orbital margin.

196 Basioccipìtal-exoccipital process ventral to occipital condyle (basioccipital plate) with parallel or ventrally convergent sides (0) or ventrally divergent sides (1) in posterior view.

197. Absence (0) or presence (1) of a smooth medial depression ventral to the basioccipital and posterior to the medial Eustachian foramen.

198. Dentary teeth series behind to alveoli 12-13 are pointed to slightly blunt (0); **or globular (1);** or molariform multicusped (2). **Modified from Salas-Gismondi et al [5], character 198.**

199. First four alveoli in the dentary are the same size or smaller than other dentary alveoli (0) or are the largest within the dentary (1).

200. Orbits longer than wide (0) or wider than long to rounded (1) late in ontogeny.

**201. From the series composed by the three most posterior premaxillary alveoli: (0) the intermediate alveolus is the biggest, or (1) anterior and intermediate alveoli are bigger, similar in size, or (2) the anterior is the biggest (modified from Salas-Gismondi et al. [5], character 201).**

**202. Frontal plate surface well ornamented with deep pits and furrows (0) or surface only little sculpted to relatively smooth (1). [New]**

**203. Retroarticular longitudinal crest** **absent (0) or present (1). [New]**

**204. Infratemporal fenestra bears an acute to straight dorsal angle, triangular shaped ITF (0); or its dorsal margin forms a gentle curve, not an angle, ovoid-shaped ITF (1). [New]**

**205. Posterior bar of supratemporal fenestra (i.e., post-temporal bar) thick (0) or thin (1). From Jouve [17], character 184.**

**206. Anterolateral margin of the orbit flush with rostral surface (0) or upturned (1). Adapted form Brochu [2], character 103.**

**Character matrix**

This data matrix is based on Brochu [2], Jouve et al. [3], and Salas-Gismondi et al. [5]. Scores for *Gavialis bengawanicus* are from Delfino & De Vos [19]. *Eosuchus lerichei* is scored after Delfino et al. [20]. Besides *Gryposuchus pachakamue*, South American gharials are represented in this matrix by *Ikanogavialis gameroi* Sill, 1970, *Piscogavialis jugaliperforatus* Kraus, 1999, *Siquisiquesuchus venezuelensis* Brochu & Rincón, 2004, *Gryposuchus colombianus* (Langston 1965), and *Gryposuchus* *croizati* Riff & Aguilera, 2008. We also include character coding of the Caribbean taxon *Aktiogavialis puertoricensis* based on Velez-Juarbe et al. [21]. *Aktiogavialis* was codable only for 13.1% of the proposed characters (i.e., 27 of 206); and considering that water abrasion affected preservation of the holotype and only specimen [21], we cautiously scored it as unknown for prootic exposure around the trigeminal foramen (i.e., character 164-?). Although *Gryposuchus neogaeus* (Burmeister, 1885) and *Gryposuchus jessei* Gürich, 1912 were included in anatomical comparisons, these taxa are not included in the current phylogenetic analyses because their tentative scorings are redundant with other *Gryposuchus* species. New material of *Piscogavialis jugaliperforatus*, comprising a well-preserved partial skull and mandibles (MUSM 439; MUSM 2528), was used to complement scores provided by Delfino et al. [20] based on the type specimen (SMNK 1282 PAL). Our matrix also includes new scorings for the non-South American taxon *Eogavialis africanus* based on direct examination of original material (AMNH 5067, AMNH 5069, AMNH 5071, AMNH 5073, AMNH 5074, AMNH 5075, SMNS 11785, SMNS 50.734).

*Bernissartia fagesii*

????? ?0??? 01111 02100 ?00?0 ?000? ??000 0?100 010?? ?0010 ?000? ????? ?10?0 ?00?0 01?1? ???00 010?0 000?0 0030? 001?? ???10 00??? ?1?00 00?00 0??01 00?0? ??001 100?0 ?0??0 ?0?00 10?0? ?00?0 0???? ????0 00?00 00?00 0000? 0?000 0??00 0?100 00000 0

*Acynodon iberoccitanus*

????? ????? ????? ????? ????? ????? ????? ????? ????? ?1010 3101? ????? ??0?? ????? 0?0?? ???00 010?0 00000 10600 00100 ??001 00??? ?0000 00010 1?00? ??200 100?1 100?1 10?00 00??0 10100 000?2 ????? ????1 ???0? ???01 00010 01100 00??1 00100 00000 0

*Iharkutosuchus makadii*

????? ????? ????? ????? ????? ????? ????? ????? ????? ?1012 3???? ????? 110?? ?00?? 10?1? ???00 01??0 00001 10610 001?0 ???01 10??? ?0000 00100 11000 01201 1001? 100?1 ?0?00 ?0??? 12??? 100?3 ????? 1???1 00000 0??11 00?10 00000 00??1 ?0200 00000 0

*Hylaeochampsa vectiana*

????? ????? ????? ????? ????? ????? ????? ????? ????? ????? ????? ????? ????? ????? ??0?? ???0? ????? ????0 ?0?10 001?0 0??01 10?0? 00000 00100 1000? 0?221 10001 110?? ??1?0 ?0000 10100 00000 0???? 1?001 00100 0?011 0001? ?0000 00??0 00??0 ?0?00 0

*Borealosuchus sternbergii*

00000 0000? 11001 0?100 10000 00101 00000 1?00? ??0?? ?0110 10000 000?0 00100 00000 10000 0??00 020?0 00000 01310 00100 01000 001?0 ?0000 01110 10001 01000 00111 100?0 00100 ?0000 00100 10100 0000? 1?001 00110 00000 00100 11000 00011 00000 00000 0

*Eothoracosaurus mississippiensis*

????? ?0??? ????? ????0 1??00 0???? ????0 0?00? ??0?? ??1?2 ???3? ????? 0???? ?00?0 21?0? ???00 130?0 00??1 02500 0010? ?0000 00??? ?0000 10100 1000? ???00 0?001 100?? ?01?? ?1000 0010? 001?0 0???? ????1 00010 00000 01001 101?1 10?1? 00000 100?0 0

*Thoracosaurus neocesariensis*

????? ?0??? ???11 1?1?0 10??? ?0011 ??0?? 0?00? ??0?? ?1122 ???3? ????? ??10? 000?0 2?0?? ???00 130?0 000?1 02500 00100 ??000 000?0 ?0000 10100 10000 ?0000 00001 110?0 0010? ?1?00 00100 00101 00000 0?001 00?10 00000 0100? ??0?1 10??0 ?00?0 ????0 0

*Eosuchus lerichei*

????? ????? ?0101 1???0 1???? ???11 ????? ??0?? ????? ?1112 ???3? ????? ????? ????? ????? ???00 120?0 00001 02500 00100 ?0010 000?? ?0000 00100 1000? 01000 10??1 110?0 00?0? 010?2 0010? 00100 ????? ????1 0??10 10100 31010 0?000 00??0 00000 00?10 0

*Eosuchus minor*

????? ?0??? 0??11 1???0 1?00? 0?01? ??000 0?000 ??0?? ?1122 ??030 0?000 0?10? 00000 2100? ???00 120?0 000?1 02500 00100 ?0000 00?00 00000 10100 10000 ?1000 00001 110?? ?0100 ?10?1 00100 001?0 100?? 1???1 01010 10100 31010 0?000 00?10 00000 00010 0

*Eogavialis africanus*

????? ???1? ????1 1???0 10??? ????? ??0?? ????? ??0?? ?11?2 2003? ????0 0?110 000?? 2101? ???00 120?0 00101 02500 00100 ?0000 00000 00000 10100 10000 01000 00001 110?0 00100 ?1001 00100 00101 00?00 0??01 01010 10000 01001 00010 10?00 11001 10010 1

*Gryposuchus colombianus*

????0 ?0??? 001?? ????0 1??00 0???? ????? ????? ??0?? ?1132 2?030 100?0 00110 00000 2100? ???00 13110 011?1 02500 00100 ?0?00 00??0 ?0000 10101 10000 01100 00000 121?0 00100 ?1001 10100 00201 00010 0?001 21010 10000 ?100? 10101 21?00 11001 21111 1

Siwalik *Gavialis*

????? ????? ????? ????? ?0??? ????? ????? ?0??? ????? ???32 ???3? ????? 0?100 000?? 2?0?? ???0? ?40?? ??10? ?2??0 ????0 ?0?0? ????0 ?0000 ?0?00 100?0 ?1??? ?0000 ?21?0 00100 0100? ?0?00 0?101 ?0??? ??00? 21010 10??? 01001 ?0111 21?00 ?1??1 ?0001 1

*Gavialis bengawanicus*

????? ????? ???11 1???? ????? ????? ????? ??1?? ????? ?1?32 ?003? ????0 00100 00000 2100? ????? ?4??? ????1 02500 00?00 ?000? ??0?? ?01?0 10??? 1??0? ??0?0 ?0000 ?21?0 0?100 01001 10100 1?101 ???0? 0?00? ?1010 10??? 01?0? 101?1 11??0 11001 ?0001 1

*Gavialis gangeticus*

02000 0000? 00111 10110 10000 00011 10000 00?00 00000 01132 20030 00000 00100 00000 21000 10000 14010 00101 02500 00100 00000 00000 00000 10100 10000 01100 00000 12100 00100 01001 10100 00101 00000 00001 21010 10000 01001 20111 21100 11001 10001 1

*Arktiogavialis puertoricensis*

????? ????? ????? ????? ????? ????? ????? ????? ????? ????? ????? ????? ????? ????? ????? ????? ????? ????? ????? ????? ????? ????? ????? ????? ?0?0? ????? ????? ?2??? ????? ??001 10100 0???1 ?00?? 0???? 210?? 1???? ???0? ????? 01??? 1???? ?00?0 ?

***Gryposuchus pachakamue***

????? ????? ????? ????? ????? ????? ????? ????? ????? ?1132 20?4? ????0 0011? 00?00 211?? ???00 120?0 01101 02500 00100 ?0000 00??? ?0000 10100 1000? 01000 00001 110?0 00000 01?01 0010? 00101 ????? 0???1 ?101? 10000 41001 10111 21000 11001 21111 1

*Gryposuchus croizati*

????? ????? ????? ????? ????? ????? ????? ????? ????? ?1132 2???? ????? ????? ????? ????? ???00 131?0 01101 02500 00100 ????? ?0??? ?0??0 ??111 1000? ?1?0? ?00?0 121?? ?010? ????1 101?? 0?201 ????? ????? 21010 1???? 41?01 10??1 21??? 11?01 211?1 1

*Piscogavialis jugaliperforatus*

????? ????? ????? ????? ????? ????? ??1?? ????0 ????? ?1132 20030 0?000 001?0 ???10 2?00? ???00 130?0 01101 02500 00100 ?0000 000?0 ?0000 10100 10000 01?00 00001 110?0 00001 01002 00100 00201 ?0?1? 0?0?1 2??1? 10000 41001 20110 2?000 1?000 10111 1

*Ikanogavialis gameroi*

????? ????? ????? ????? ????? ????? ????? ????? ????? ?1132 ????? ????? 0?10? ??0?0 2?00? ????? ?30?? 0?1?1 02500 0010? ???00 0???? ??0?0 10??0 10??? ??000 ??000 ?11?? ?0?0? ????2 10100 00201 ????? ????? 21010 10??0 ?1?01 20?10 2???0 11?01 ?01?1 1

*Siquisiquesuchus venezuelensis*

????? ????? ????? ????? 1???? ????? ????? ????? ????? ?1132 ???3? 0???? ????? ????0 ????? ????? 1?0?? 0???1 02500 001?? ???0? ????? ?0000 10??0 1???? ????? ??000 ?20?? ?0??? ?1?0? ?010? 002?1 ????? ???0? 21010 10??0 41?0? 20?10 2??00 ?0?00 ?01?1 1

*Argochampsa krebsi*

????? 0?00? 0?11? 1???? ????? ?0??? ????? ???0? ????? ?11?2 ????? ????? ????? ????? ????? ????? 130?0 00001 02500 00100 ??0?? 0?1?? ?00?0 10??? 1000? ??000 10111 110?? ?0000 0?001 1010? 00201 ?0??? 0???? ???1? 1?000 01000 200?0 01??0 11?00 10??0 1

*Pristichampsus vorax*

????0 ?0??? 01001 001?0 1?000 00111 ??010 0?10? ??1?? ?1110 ?000? ????0 ??100 0001? ??0?? ???21 010?0 000?0 00300 01100 ?0000 00??0 ?0000 00100 10001 01000 00111 110?? ?0100 00100 00100 10100 0???0 1??11 00110 00000 20110 01000 00??1 000?0 00000 0

*Planocrania hengdongensis*

????? ????? ????? ????? 1???? ????? ????? ????? ????? ?1110 ???0? ????? ??1?? ??0?0 ??0?? ???20 010?0 ????? 01300 ??1?? ??0?0 00??? ?0??? ?0100 1???? ???0? 0?1?1 100?? ????? ?0?0? 00100 ?0100 ????? 1???1 ???10 ?1000 1???? ????? ????? ??0?0 000?0 0

*Leidyosuchus canadensis*

????0 ?0??? ????1 ????0 10000 011?1 ??10? 0?11? ?11?? ?0110 ?0000 0?0?0 1?110 00001 1101? ???00 010?0 00000 00300 00100 00010 00100 01000 01110 10001 01000 10111 100?0 10100 10010 00100 10100 00000 1?001 00110 01000 10100 11000 00?01 00000 00000 0

*Diplocynodon ratelii*

????? ?0??? ???00 0???0 10?00 ?1111 00140 0?10? ?21?? ?0100 1101? ????0 11100 0001? ?101? ???00 120?0 00010 12300 00100 01010 00??0 ?0000 00111 10001 01000 10111 100?? 10100 10000 00101 10100 00000 1?101 00110 01000 10110 01000 00011 00000 00000 0

*Brachychampsa montana*

10101 1001? 1100? ??000 1??00 01111 00?00 0?103 111?? ?1110 0101? ????0 1110? 00001 11010 0??00 110?0 00311 10100 00100 ?0010 001?0 ?0100 00011 11001 01210 11111 100?1 10200 ?0111 00101 10103 00010 1?001 00110 01000 10110 01000 00011 00100 00000 0

*Alligator mississippiensis*

10101 1001? 01001 00000 10110 11111 00110 00112 10110 01100 01120 10010 11200 01011 11010 01100 10000 10010 10200 00100 00010 00111 00000 00011 11002 01101 11111 11001 10200 10112 10102 10102 00011 11001 00110 01000 10110 01000 00011 00000 00000 0

*Navajosuchus mooki*

????? ?0?1? ????? 0???0 ?00?? ?1111 ??1?? 0?111 111?? ?1111 0010? ????0 1?11? ?00?? 110?? ???00 010?0 00210 10200 001?? ?0?10 00??0 ???00 ?00?1 1100? 01110 11111 100?? ?020? ?0112 ?0102 10100 0???? 1??01 0??10 01000 10110 0?000 000?1 00100 00000 0

*Purussaurus neivensis*

101?1 00?1? 00001 0?0?? 1???? 011?? ????? 0?11? ??1?? ?1100 ??1?1 010?1 11201 10001 1001? ???00 110?0 00020 10000 01110 ?0010 001?0 ?0000 00011 11011 11021 01111 110?1 10201 ?0112 001?2 10104 00010 1??01 0??10 21000 10?10 01000 00?01 00010 00000 0

*Mourasuchus atopus*

10??1 00?1? 00?01 0?00? 1?10? 011?? ??130 0?11? ??1?? ?1102 ?112? ????0 1110? 10001 1000? ???00 120?1 00001 10500 01100 ?0010 011?? ?0000 00011 11011 ?1021 0?111 111?1 10?00 10??2 111?? 12104 0???? 1???1 00110 ?1000 10110 210?0 00??? ?0?10 00000 1

*Caiman crocodilus*

10111 1001? 10001 00000 10101 11111 00110 00111 22111 01100 11121 01011 10101 10201 10010 11100 11000 00020 11200 00100 00010 00110 01000 00011 11011 11110 11111 11001 10201 10112 01112 10104 00010 11001 00110 21000 10110 01000 00011 00000 00000 0

*Paleosuchus trigonatus*

10011 1111? 01001 01000 10001 11111 21130 00111 32112 01100 11222 11111 11101 10201 10010 11110 11000 01010 10200 00100 00010 00110 00100 01111 11011 11000 11111 11011 10201 10112 121?2 10103 00010 11001 00110 21000 10110 01000 00011 00000 00000 0

*Culebrasuchus mesoamericanus*

????? ????? ????? ????? ????? ????? ????? ????? ????? ?1100 ???2? ????0 ??20? ????? ????? ???10 ????0 ????? ?000? 0?1?? ????? ????0 ?0??? ????? ????? ????? ??1?1 ?00?? ????? ????2 10?0? ??1?4 ????? ????1 ???1? 0???? ?0??? ????0 00??? 00000 000?0 0

*Globidentosuchus brachyrostris*

????? ????? ????? ????0 1???? ????? ????? ????? ????? ?1110 0??0? 1???0 ?1101 10311 1001? ???00 1???0 00??0 1?200 00??? ?1?10 ?0??? ?1?0? ??0?1 1???? ?1020 0?1?1 100?? ????0 ?0??2 011?? 101?4 ????? ????1 ???1? ????0 ?0?1? 010?0 00??? 00100 00000 0

*Gnatusuchus pebasensis*

????? ????? ????? ????? ????? ????? ????? ????? ????? ?1020 ?1200 0?011 11101 00001 1001? ???02 110?0 10010 10000 001?? ?0010 ?0??? ?1??? ??11? 10011 01?1? 1???1 100?? ?0?0? 1?012 0111? 10104 ????? 1??01 00110 21001 10110 01000 00011 00101 00000 0

*Crocodylus niloticus*

10100 0001? 10101 00010 11100 11111 20120 00111 20110 11100 11010 10001 01100 01110 20111 00100 11000 00010 02100 00100 11000 00110 10010 00110 10010 01000 00111 11000 01011 00012 00100 10100 01110 10111 10011 00000 30110 01000 00011 00000 00000 0

*Crocodylus acutus*

00100 0001? 10101 10010 11100 11111 20120 00110 20110 11100 11010 10001 01100 01110 20111 00100 11000 00010 02101 00100 11000 00110 10010 00110 10000 01100 00111 11000 01011 00012 00100 10100 01110 10111 10011 00000 30110 01000 00011 00000 00000 0

*Crocodylus acer*

????? ????? ????? ????? ????? ????? ????? ????? ????? ????? ????? ????? ????? ????? ????? ???00 110?0 000?1 02100 00100 ??000 001?? ?0001 00?10 10000 01000 10111 100?0 02?01 ?00?2 00100 10100 01??0 ??111 00010 00000 30?10 01000 00?11 00??0 0?000 0

*Crocodylus affinis*

00100 1001? 10001 00001 11000 01111 00110 0?10? ??1?? ?1110 11010 10000 01100 00000 20110 0??00 110?0 00010 01100 00100 ?0100 00??0 ?0001 00110 10000 01000 00111 100?0 ?010? ?0012 00100 10100 01??0 ???11 00010 00000 30?10 01000 000?1 00??0 0??00 0

*Tomistoma schlegelii*

02100 0001? 00101 00010 11000 11111 10110 00101 30110 11122 ?1040 00001 00100 00000 20100 00100 12000 00011 02100 00101 01000 00110 10001 00000 10000 01100 00111 11000 00110 00012 10100 10100 01100 10111 10010 00000 30110 01000 00011 00000 00000 0

*Thecachampsa americana*

????? ????? ????1 ????0 1?000 ?1111 ??110 0?00? ??1?? ?1122 ???4? ????0 0010? 0031? ?010? ???00 120?0 00011 02100 00100 ?1000 00??? ?0000 10000 10000 ??100 0?111 110?0 00?10 01012 00100 10102 ???0? ???11 10010 0?200 301?0 01000 00??? 00000 00010 0

*Kentisuchus spenceri*

????? ?0??? ????? ????? 1???? ????? ????? ????? ????? ??1?0 ???4? ????0 ??100 0?110 ??11? ???00 110?0 000?1 02100 00100 ???00 00??0 ?0100 10100 100?0 01100 0?111 110?? ????0 ?0??2 0010? 10100 ????? ??111 ?0?10 0?0?0 30?10 0?0?0 00??1 00??0 000?0 0

*Asiatosuchus germanicus*

001?0 ?0?1? 00101 0?010 1?000 ?1111 ??1?? 0???? ??1?? ?1110 1000? ????0 01100 00??0 101?0 0??00 010?0 000?0 00100 0010? ??100 00??0 ?0001 0???0 10000 01000 0?111 100?? ?0100 ?0011 10100 10100 0???? ???11 00?10 00000 30?10 010?0 0?001 ????0 000?0 0

**(2) Additional morphometric data and results**

**Landmark definitions.**

1. Anteriormost point of the frontal on the sagittal axis.

2. Fronto-parietal suture on the sagittal axis.

3. Right postorbital-squamosal suture on the lateral margin of skull table.

4. Left postorbital-squamosal suture on the lateral margin of skull table.

5. Right antero-lateral corner of skull table.

6. Left antero-lateral corner of skull table.

7. Right contact of postorbital-frontal suture with the orbital margin.

8. Left contact of postorbital-frontal suture with the orbital margin.

9. Right contact of frontal-prefrontal suture with orbital margin.

10. Left contact of frontal-prefrontal suture with orbital margin.

11. Right contact of prefrontal-lacrimal suture with the orbital margin.

12. Left intersection of prefrontal-lacrimal suture with the orbital margin.

13. Right frontal-prefrontal-nasal junction.

14. Left frontal-prefrontal-nasal junction.

15. Right anteriormost point of prefrontal.

16. Left anteriormost point of prefrontal.

17. Right anteriormost point of jugal.

18. Left anteriormost point of jugal.

19. Right anteriormost point of lacrimal.

20. Left anteriormost point of lacrimal.

**Crocodylian skull material studied in the morphometric analysis**

1. *Eosuchus* cf. *minor* ANSP 10079

2. *Eosuchus* *lerichei* IRSNB 1740

3. *Thoracosaurus macrorhynchus*, MNHN 1902-22

4. *Eogavialis africanus*, IMGP-UT

5. *Argochampsa krebsi*, OCP DEK-GE 1201

6. *Gryposuchus pachakamue*, sp. nov., MUSM 1981

7. *Gryposuchus colombianus*, IGM 184696

8. *Ikanogavialis gameroi*, MCNC 143-72V

9. *Piscogavialis jugaliperforatus*, SMNK 1282 PAL

10. cf. *Piscogavialis* sp., MUSM 1997

11. *Gavialis browni* (Siwaliks *Gavialis*), AMNH 6279

12. *Gavialis gangeticus*, MNHN A5321

13. *Gavialis bengawanicus*, DMR-KS-201202-1

14. *Borealosuchus sternbergii*, USNM 6533

15. *Leidyosuchus canadensis* NMC 8942

16. *Alligator mississippiensis* UF 10941

17. *Caiman crocodilus* UF 80913

18. *Paleosuchus trigonatus*, MUSM DPV CR1

19. *Crocodilus niloticus* MNHN

20. *Crocodilus acutus* UF 49953

21. *Tomistoma schlegelii*, MNHN A5311

22. *Thecachampsa americana* AMNH 5663

Institutional Abbreviations. AMNH: American Museum of Natural History, New York, USA. ANSP: Academy of Natural Sciences, Philadelphia, USA. DMR-KS: Khok Sung Collection, Department of Mineral Resources, Bangkok, Thailand. IGM: INGEOMINAS, Bogotá, Colombia. IRSNB: Institut Royal des Sciences Naturelles de Belgique, Brussels, Belgique. MCNC: Museo de Ciencias Naturales de Caracas, Caracas, Venezuela. MNHN: Muséum national d’Histoire naturelle, Paris, France. MUSM, Vertebrate Paleontology Collection of the Natural History Museum of San Marcos University, Lima, Peru. NMC: Canadian Museum of Nature, Ottawa, Canada. OCP: Office Chérifien des Phosphates, Khouribga, Morocco. SMNK: Staatliches Museum für Naturkunde Karlsruhe, Karlsrhue, Germany. UF: Florida Museum of Natural History - University of Florida, Gainesville, USA. USNM: United States National Museum, Smithsonian Institution, Washington, USA.

**Procrustes superimposition analysis [22]. Average shape:**

**Lmk. Axis 1 (x) Axis 2 (y)**

1 0.17274774 0.00000000

2 -0.25346838 0.00000000

3 -0.29071048 -0.22922749

4 -0.29071048 0.22922749

5 -0.17876069 -0.20004794

6 -0.17876069 0.20004794

7 -0.15485176 -0.11980901

8 -0.15485176 0.11980901

9 -0.06315062 -0.07431625

10 -0.06315062 0.07431625

11 0.00630870 -0.11563227

12 0.00630870 0.11563227

13 0.06566544 -0.02265320

14 0.06566544 0.02265320

15 0.17356595 -0.04997391

16 0.17356595 0.04997391

17 0.17384811 -0.12972111

18 0.17384811 0.12972111

19 0.30844567 -0.05506090

20 0.30844567 0.05506090

Procrustes sums of squares: 0.7263382866828477

Tangent sums of squares (symmetric component): 0.6888259374667368

Tangent sums of squares (asymmetry component): 0.007601511570988219

**Principal Component Analysis (PCA): CovMatrix, newDataset, Symmetric component [22,23]**

Eigenvalues % Variance Cumulative %

1. 0.01475838 44.993 44.993

2. 0.00831802 25.359 70.352

3. 0.00359941 10.973 81.326

4. 0.00156568 4.773 86.099

5. 0.00125842 3.837 89.935

6. 0.00096193 2.933 92.868

7. 0.00062272 1.898 94.766

8. 0.00043773 1.334 96.101

9. 0.00036539 1.114 97.215

10. 0.00033475 1.021 98.235

11. 0.00020617 0.629 98.864

12. 0.00016015 0.488 99.352

13. 0.00009950 0.303 99.656

14. 0.00005683 0.173 99.829

15. 0.00002574 0.078 99.907

16. 0.00001716 0.052 99.960

17. 0.00000969 0.030 99.989

18. 0.00000355 0.011 100.000

Total variance: 0.03280124

Variance of the eigenvalues: 0.0000136694038

Eigenvalue variance scaled by total variance: 0.01270

Eigenvalue variance scaled by total variance and number of variables: 0.24214

Statistical analysis for fluctuating asymmetry

This analysis takes into account the object symmetry in the data:

Classifiers used for the Procrustes ANOVA:

Individuals: Taxa

Centroid size:

Effect SS MS df F P (param.)

Individual 2052.454831 97.735944 21

Shape, Procrustes ANOVA:

Effect SS MS df F P (param.)

Individual 0.68882594 0.0018222908 378 95.93 <.0001

Side 0.00042081 0.0000233781 18 1.23 0.2329

Ind * Side 0.00718071 0.0000189966 378

Shape, MANOVA tests of effects:

Symmetric component of shape variation:

Effect Pillai tr. P (param.)

Note: the test for 'Individual' used the symmetric component of the residual as the 'error' effect.

Asymmetry component of shape variation:

Effect Pillai tr. P (param.)

Side 0.78 0.6694

Directional asymmetry vector:

Lmk. Axis 1 (x) Axis 2 (y)

1 0.00000000 -0.00067460

2 0.00000000 -0.00200719

3 0.00091023 -0.00064757

4 -0.00091023 -0.00064757

5 0.00035812 0.00091065

6 -0.00035812 0.00091065

7 0.00093445 0.00156955

8 -0.00093445 0.00156955

9 -0.00007215 0.00051097

10 0.00007215 0.00051097

11 0.00019921 0.00019665

12 -0.00019921 0.00019665

13 0.00024572 0.00020511

14 -0.00024572 0.00020511

15 -0.00031841 -0.00043032

16 0.00031841 -0.00043032

17 -0.00028206 -0.00016309

18 0.00028206 -0.00016309

19 0.00053116 -0.00081105

20 -0.00053116 -0.00081105

**Phylogenetic mapping**

Phylogenetic tree notation (Newick format): ((1,2,(3,(4,(5,(((6,7),(8,(9,10))),(11,12,13)))))),(14,((15,(16,(17,18))),((19,20),(21,22)))))

Dataset: PC scores, CovMatrix, newDataset, Symmetric component [23].

Data type: PC scores.

Method: unweighted squared-change parsimony.

The tree is rooted.

Tree length: 0.23046511

**References**

1. Brochu CA. Phylogenetic relationships of *Necrosuchus ionensis* Simpson, 1937 and the early history of caimanines. Zool J Linn Soc. 2011; 163: 228-256. doi:10.1111/j.1096-3642.2011.00716.x
2. Brochu CA. Phylogenetics, taxonomy, and historical biogeography of Alligatoroidea. Soc Vertebr Paleontol Mem. 1999; 6: 9-100. doi:10.1080/02724634.1999.10011201
3. Jouve S, Bardet N, Jalil N-E, Suberbiola XP, Bouya B, Amaghzaz M. The oldest African crocodylian: phylogeny, paleobiogeography, and differential survivorship of marine reptiles through the Cretaceous-Tertiary boundary. J Vertebr Paleontol. 2008; 28: 409-421. doi:10.1671/0272-4634(2008)28[409:TOACPP]2.0.CO;2
4. Riff D, Aguilera OA. The world’s largest gharials *Gryposuchus*: description of *G*. *croizati* n. sp. (Crocodylia, Gavialidae) from the Upper Miocene Urumaco Formation, Venezuela Paläont Z. 2008; 82: 178-195. doi:10.1007/BF02988408
5. Salas-Gismondi R, Flynn JJ, Baby P, Tejada-Lara JV, Wesselingh FP, Antoine P-O. A Miocene hyperdiverse crocodylian community reveals peculiar trophic dynamics in proto-Amazonian mega-wetlands. Proc R Soc B 2015; 282: 20142490. doi:10.1098/rspb.2014.2490
6. Norell MA. PhD. thesis, Yale University, 1988.
7. Norell MA. The higher level relationship of the extant Crocodylia. J Herpetol. 1989; 23: 325-335.
8. Clark JM. Patterns of evolution in Mesozoic Crocodyliformes. In: Fraser NC, Sues H-D, editors. In the Shadow of the Dinosaurs: Early Mesozoic Tetrapods. New York: Cambridge University Press; 1994. pp. 84-97.
9. Benton MJ, Clark JM. Archosaur phylogeny and the relationship of the Crocodylia. In: Benton MJ, editor. The Phylogeny and Classification of the Tetrapods 1. Oxford: Clarendon Press, 1988. pp. 295-338.
10. Norell MA, Clark JM. A reanalysis of *Bernissartia fagesii*, with comments on its phylogenetic position and its bearing on the origin and diagnosis of the Eusuchia. Bull Inst R Sci Nat Belg. 1990; 60: 115-128.
11. Willis PMA. *Trilophosuchus rackhami* gen et sp. nov., a new crocodilian from the early Miocene limestones of Riversleigh, northwestern Queensland. J Vertebr Paleontol. 1993; 13: 90-98.
12. Salisbury SW, Molnar RE, Frey E, Willis PMA. The origin of modern crocodyliforms: new evidence from the Cretaceous of Australia. Proc R Soc London Ser B 2006; 273: 2439-2448. doi:10.1098/rspb.2006.3613
13. Hua S, Jouve S. A primitive marine gavialoid from the Paleocene of Marocco. J Vertebr Paleontol. 2004; 24: 341-350. doi:10.1671/1104
14. Buscalioni AD, Sanz JL, Casanovas ML. A new species of the eusuchian crocodile *Diplocynodon* from the Eocene of Spain. Neues Jahrb Geol Palaeontol Abh. 1992; 187: 1-29.
15. Ösi A, Clark JM, Weishampel DB. First report on a new basal eusuchian crocodyliform with multicusped teeth from the Upper Cretaceous (Santonian) of Hungary. Neues Jahrb Geol Palaeontol Abh. 2007; 243: 169-177.
16. Buscalioni AD, Ortega F, Weishampel DB, Jianu CM. A revision of the Crocodyliform *Allodaposuchus precedens* from the upper Cretaceous of the Hateg basin, Romania. Its relevance in the phylogeny of the Eusuchia. J Vertebr Paleontol. 2001; 10: 244-254. doi:10.1671/0272 4634(2001)021[0074:AROTCA]2.0CO;2
17. Jouve S. PhD. thesis, Museum national d’Histoire naturelle, Paris, 2004.
18. Brochu CA. Alligatorine phylogeny and the status of *Allognathosuchus* Mook, 1921. J Vertebr Paleontol. 2004; 14: 857-873. doi:10.1671/0272-4634(2004)024[0857:APATSO]2.0.CO;2
19. Delfino M, De Vos J. A revision of the Dubois Crocodylians, *Gavialis* *bengawanicus* and *Crocodylus* *ossifragus*, from the Pleistocene *Homo erectus* beds of Java. J Vertebr Paleontol. 2010; 30: 427-441.
20. Delfino M, Piras P, Smith T. Anatomy and phylogeny of the gavialoid crocodylian *Eosuchus lerichei* from the Paleocene of Europe. Acta Pal Polon. 2005; 50: 565-580.
21. Velez-Juarbe J, Brochu CA, Santos H. A gharial from the Oligocene of Puerto Rico: transoceanic dispersal in the history of a non-marine reptile. Proc R Soc B 2007; 274: 1245-1254. doi:10.1098/rspb.2006.0455
22. Adams DC, Otarola-Castillo E. Geomorph: an R package for the collection and analysis of geometric morphometric shape data. Methods Ecol Evol. 2014; 4: 393-399.
23. Klingenberg CP. MorphoJ: an integrated software package for geometric morphometrics. Mol Ecol Resour. 2011; 11: 353-357.
