# Supplementary figures and images for: A New 13 Million Year Old Gavialoid Crocodylian from Proto-Amazonian Mega-Wetlands Reveals Parallel Evolutionary Trends in Skull Shape Linked to Longirostry
Source: PLoS One. 2016 Apr 20;11(4):e0152453. doi: 10.1371/journal.pone.0152453 (PMC4838223; doi:10.1371/journal.pone.0152453)

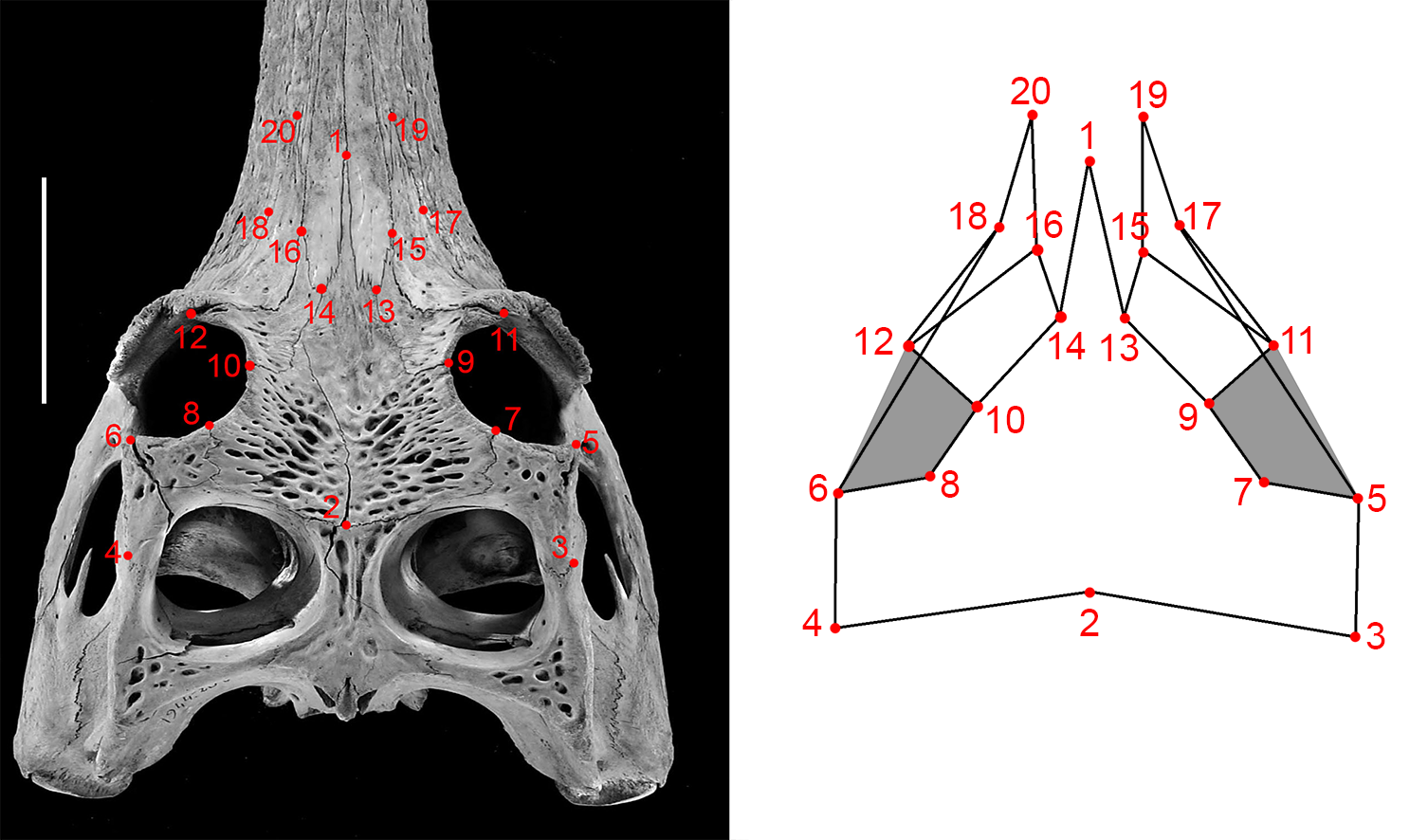

Supplement: S1 Fig — Landmarks are labeled from 1 to 20. Gray area symbolizes orbits defined by landmarks 5-7-9-11 and 6-8-10-12 on the right and left sides, respectively. (TIF) [file pone.0152453.s002.tif]

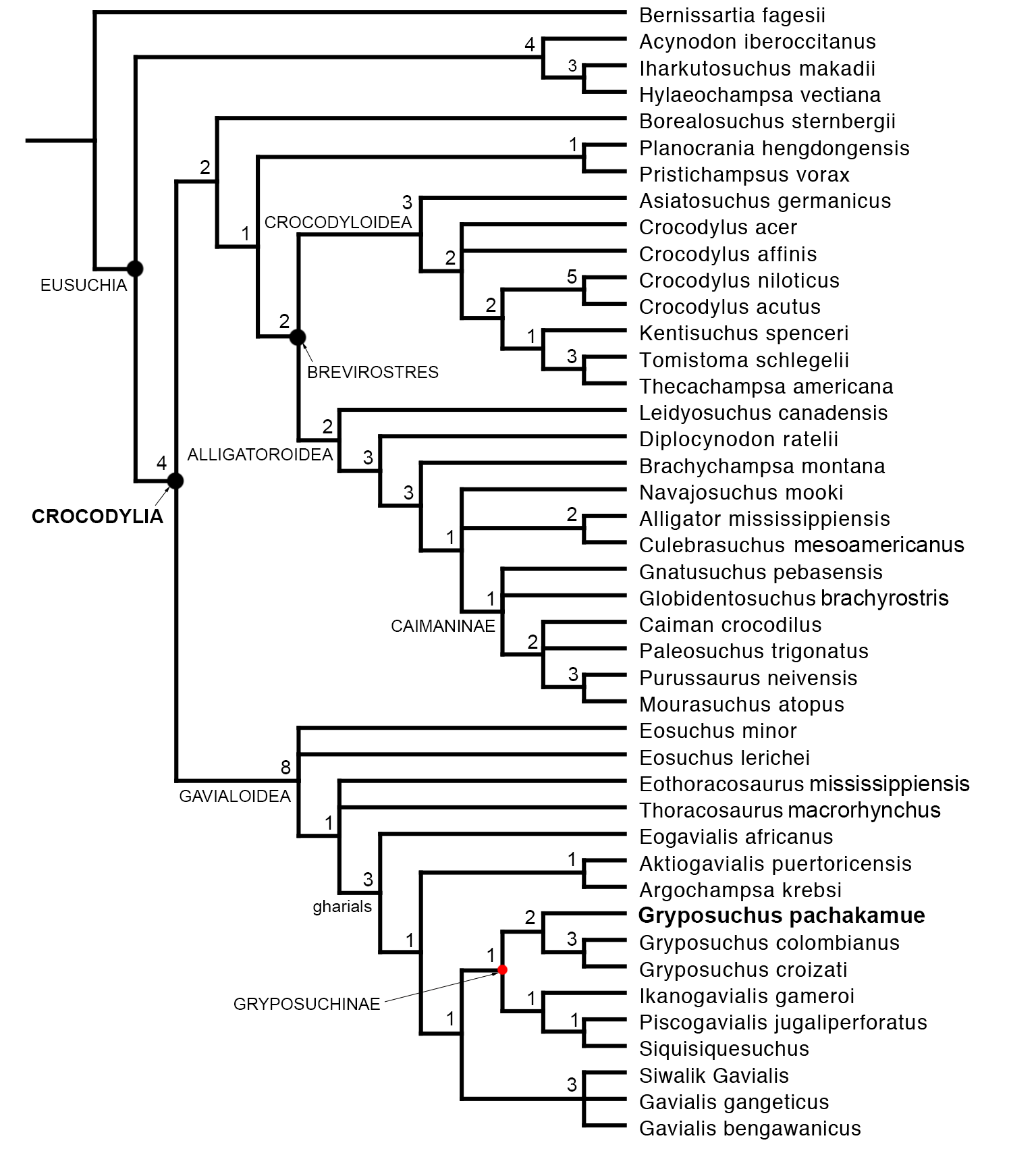

Supplement: S2 Fig — Second approach after removing character state 137–2 and character 138. Numbers at nodes indicate Bremer support values. (TIF) [file pone.0152453.s003.tif]
